# A Nonlinear Three-Mode Model of Solar Activity Based on Coupled Oscillators and Nonlinear Energy Transfer

**DOI:** 10.3390/e28101114

**Published:** 2026-10-12

**Authors:** Vitaliy Kapytin, Alexey Andreyev, Beibit Zhumabayev, Anastasiya Yanina, Yekaterina Chsherbulova, Stanislav Utebayev

**Affiliations:** 1 Ionosphere Institute, Almaty 050020, Kazakhstan; alexey.andreyev@ionos.kz (A.A.); beibit.zhu@mail.ru (B.Z.); chsherbulova@ionos.kz (Y.C.); utebayev.s@ionos.kz (S.U.); 2 Institute of Plant Biology and Biotechnology, Almaty 050040, Kazakhstan; akapytina@gmail.com

**Keywords:** solar activity, reduced-order model, nonlinear dynamics, Van der Pol oscillator, Duffing oscillator, structural energy exchange, d-index, solar cycle, magnetic energy release, self-organization

## Abstract

Solar activity exhibits a dominant decadal timescale embedded in nonlinear multiscale dynamics. We present a minimal autonomous three-mode hybrid model to investigate whether such a timescale can arise from internal nonlinear energy exchange rather than external periodic forcing or direct inheritance of a prescribed modal period. The model couples a slow internal reservoir, a tachocline-associated transfer mode, and a nonlinear magnetic subsystem described by Duffing and Van der Pol–Duffing dynamics. Magnetic-energy release is implemented through a threshold-triggered reset, while a D-index-based structural energy-exchange index characterizes relative magnetic-energy changes. The system develops a persistent bounded hybrid regime with a dominant period of approximately 10 yr. Initial-condition ensembles and variations in the intrinsic modal periods demonstrate that this timescale does not simply track the prescribed 11 yr tachocline period. Parameter studies identify the release depth and magnetic recovery rate as stronger controls, supporting an accumulation–release mechanism of timescale selection. Release events exhibit organized recurrence and significant tachocline-phase preference, with localized event skipping separating spectral and release timescales. Saltation-based Lyapunov analysis reveals no robust positive largest exponent for the baseline regime over the investigated finite-time horizons. These results demonstrate that nonlinear energy exchange combined with threshold-mediated accumulation and release can autonomously generate a robust decadal collective timescale in a reduced nonlinear system.

## 1. Introduction

Solar activity exhibits variability over a broad hierarchy of temporal scales. Its most prominent long-term manifestation is the approximately 11-year Schwabe cycle, accompanied by substantial cycle-to-cycle variations in amplitude and duration, long-term modulation, intermittent episodes of reduced activity, and shorter quasi-periodic components [1,2,3,4,5,6]. Quasi-periodic variability is also observed on shorter timescales in flare-related emission [7]. Multifractality and multiscale organization have been identified in solar magnetic fields and sunspot records [8,9,10,11]. The solar cycle should therefore be regarded not as a strictly periodic process governed by a single frequency, but as a dominant timescale embedded in a complex nonlinear system.

The solar magnetic cycle is generally attributed to dynamo processes involving differential rotation, convection, meridional circulation, magnetic induction, and nonlinear feedback [6,12,13,14,15,16,17,18]. Solar-dynamo models range from low-dimensional phenomenological descriptions to spatially resolved mean-field and three-dimensional magnetohydrodynamic (MHD) models [12,18,19]. Reduced-order approaches complement these models by isolating specific dynamical mechanisms through a small number of effective degrees of freedom. Examples include time-delay dynamos [20], low-order models of cyclic variability and grand minima [21], and Duffing or Van der Pol–Duffing representations of solar-cycle dynamics [22,23,24,25]. More broadly, self-organization, MHD oscillations, and nonlinear plasma dynamics provide complementary frameworks for interpreting multiscale and nonstationary behavior in solar and space plasmas [26,27,28]. Nonlinear-oscillator theory provides a compact framework for representing self-sustained dynamics, amplitude regulation, nonlinear frequency shifts, and interactions among effective dynamical modes [29,30,31].

A central issue for reduced models is whether a solar-like timescale is genuinely generated by collective dynamics or merely inherited from prescribed modal periods or external forcing. For example, externally synchronized dynamo models can generate cycle organization through periodic modulation; Stefani et al. [32] considered an α-effect modulated by an assumed planetary tidal forcing with an approximately 11.07-year periodicity. In an autonomous model, by contrast, the characteristic timescale must arise from internal dynamics. Demonstrating a spectral peak near the Schwabe period is therefore insufficient: its persistence should also be tested against variations in the intrinsic modal periods and governing parameters.

The present study addresses this problem from an energetic perspective. Motivated by the mechanics of structured systems [33,34,35,36,37,38], three collective modes are treated as interacting subsystems capable of exchanging energy. The coupling terms represent effective channels of energy redistribution rather than literal mechanical interactions between specific solar layers. A structural energy-exchange index, motivated by the energy-rate interpretation underlying the D-index framework [39], characterizes the relative rate of magnetic-energy accumulation or depletion. Magnetic-energy release is represented by a threshold-triggered discrete reset, reflecting phenomenologically the general picture of gradual energy accumulation followed by rapid release [40,41,42,43,44].

We investigate a minimal autonomous hybrid system consisting of a slow internal reservoir, a tachocline-associated energy-transfer mode, and a nonlinear magnetic subsystem. The generalized variables and modal energies are effective quantities rather than direct representations of individual solar layers or absolute physical energies. The central hypothesis is that nonlinear inter-mode energy exchange combined with threshold-mediated accumulation, release, and recovery can generate a persistent decadal collective timescale without external periodic forcing and without this timescale being simply inherited from a prescribed intrinsic modal period.

To test this hypothesis, spectral analysis is combined with systematic variations in the intrinsic periods, initial-condition ensembles, and parameter-space robustness tests. Release-event statistics, tachocline-phase organization, energy-balance diagnostics, and conservative-limit validation are examined independently, while the dynamical regime is characterized using a hybrid Lyapunov analysis incorporating the saltation matrix at threshold resets.

The results reveal a persistent bounded hybrid regime with an approximately decadal collective timescale that remains robust under substantial variations in the prescribed intrinsic periods and is controlled more strongly by parameters governing magnetic-energy release and recovery. Localized event skipping further shows that spectral organization and release recurrence are related but distinct properties. The model is therefore interpreted as a minimal phenomenological demonstration that nonlinear inter-mode energy exchange combined with threshold-mediated accumulation–release dynamics can autonomously select a robust collective timescale, rather than as a quantitatively calibrated or unique model of the solar dynamo.

## 2. Mathematical Model and Methods

### 2.1. Three-Mode Hybrid Model

We consider an autonomous reduced-order model consisting of three interacting nonlinear modes: a slow internal mode $x_{1}$, a tachocline-related mode $x_{2}$, and an active magnetic mode $x_{3}$.

The generalized coordinates $x_{1}$, $x_{2}$, and $x_{3}$ represent effective collective modes rather than directly measurable solar observables. They describe, respectively, a slow internal mode, a tachocline-associated energy-transfer mode, and a collective magnetic subsystem.

The corresponding energies $E_{i}$ are therefore expressed in normalized model units. Conversion to absolute physical units would require an independent calibration of the generalized coordinates and modal energy scales, which is beyond the present reduced model. Accordingly, the analysis focuses on relative energy redistribution, release fractions, characteristic timescales, and their robustness rather than on the absolute solar energy budget.

Introducing $v_{i}={\dot{x}}_{i}$, the state vector is

(1) $$y={\left(x_{1},x_{2},x_{3},v_{1},v_{2},v_{3}\right)}^{T}.$$

Between discrete release events, the system evolves according to the six-dimensional autonomous equations:

(2) $$\begin{array}{l} {\dot{x}}_{1}=v_{1},\\ {\dot{v}}_{1}=-\gamma_{1}v_{1}-\omega_{1}^{2}x_{1}-\alpha_{1}x_{1}^{3}+k_{12}(x_{2}-x_{1}),\\ {\dot{x}}_{2}=v_{2},\\ {\dot{v}}_{2}=-\gamma_{2}v_{2}-\omega_{2}^{2}x_{2}-\alpha_{2}x_{2}^{3}+k_{12}(x_{1}-x_{2})+k_{23}(x_{3}-x_{2})+2\beta x_{2}x_{3},\\ {\dot{x}}_{3}=v_{3},\\ {\dot{v}}_{3}=-\mu (x_{3}^{2}-1)v_{3}-\omega_{3}^{2}x_{3}-\alpha_{3}x_{3}^{3}+k_{23}(x_{2}-x_{3})+\beta x_{2}^{2}. \end{array}$$

For compact notation, the continuous dynamics defined by Equation (2) are hereafter written as

(3) $$\dot{y}=F(y),$$

where $F:{\mathbb{R}}^{6}\to {\mathbb{R}}^{6}$ denotes the autonomous vector field given by the right-hand side of Equation (2).

The intrinsic angular frequencies are defined as $\omega_{i}=\frac{2\pi }{T_{i}},$ with baseline modal periods $T_{1}$ = 22 yr, $T_{2}$ = 11 yr, $T_{3}$ = 5 yr.

These periods denote the characteristic timescales of the slow internal, tachocline-related, and magnetic modes, respectively. Importantly, $T_{i}$ specifies only the intrinsic timescales of the uncoupled modes; the period of the coupled hybrid system is not prescribed by any single $T_{i}$ but emerges from the combined effects of nonlinear coupling, magnetic self-excitation, and threshold-triggered energy release.

The coefficients $\gamma_{1}$ and $\gamma_{2}$ describe linear damping of the first two modes, whereas $\alpha_{i}$ determines the strength of the cubic Duffing nonlinearity and hence the amplitude-dependent restoring response of each mode [29,30]. The parameter μ controls the Van der Pol-type nonlinear self-excitation and dissipation of the magnetic mode: the term $-\mu (x_{3}^{2}-1)v_{3}$ provides effective amplification at small $\left|x_{3}\right|$ and dissipation at larger amplitudes [30,31]. Such combinations of nonlinear restoring, dissipative, and coupling terms constitute a standard framework for reduced nonlinear dynamical systems [30,31].

The linear coupling terms proportional to $k_{12}$ and $k_{23}$ describe reciprocal linear interactions between the slow and tachocline-related modes and between the tachocline-related and magnetic modes, respectively. The coefficient β controls the reciprocal nonlinear interaction between the tachocline-related and magnetic modes generated by the potential:

(4) $$V_{\beta }(x_{2},x_{3})=-\beta x_{2}^{2}x_{3}.$$

which gives the reciprocal forces $2\beta x_{2}x_{3},$ and $\beta x_{2}^{2}$.

No external periodic forcing is applied. The continuous dynamics are supplemented by a threshold-triggered reset when the magnetic energy $E_{3}$ reaches $E_{\mathrm{crit}}$ from below.

$E_{\mathrm{crit}}$ defines the magnetic-energy threshold for a discrete release event, while 0<η<1 specifies the fraction of the magnetic-mode energy removed at each threshold crossing.

At each event,

(5) $$E_{3}^{+}=(1-\eta )E_{3}^{-}.$$

Thus, the model combines continuous nonlinear energy exchange with discrete magnetic-energy release.

### 2.2. Inter-Mode Coupling and Energy Balance

Energy exchange between the three modes is described by reciprocal linear and nonlinear interactions. The corresponding interaction potentials are

(6) $$\begin{array}{l} V_{12}=\frac{k_{12}}{2}{\left(x_{2}-x_{1}\right)}^{2},\\ V_{23}=\frac{k_{23}}{2}{\left(x_{3}-x_{2}\right)}^{2},\\ V_{\beta }(x_{2},x_{3})=-\beta x_{2}^{2}x_{3}. \end{array}$$

The latter generates the nonlinear coupling terms

(7) $$\begin{array}{l} -\frac{\partial V_{\beta }}{\partial x_{2}}=2\beta x_{2}x_{3},\\ -\frac{\partial V_{\beta }}{\partial x_{3}}=\beta x_{2}^{2}. \end{array}$$

ensuring an energetically reciprocal interaction between the tachocline-related and magnetic modes.

It should be emphasized that $x_{i}$ is a generalized modal coordinate rather than an energy variable itself. Its sign characterizes the phase of the corresponding collective mode and therefore does not imply a positive or negative energy of the subsystem. The energetic interpretation of each mode is introduced below through the corresponding modal energy, defined as a function of the generalized coordinate and velocity.

The effective energy of each mode is defined as

(8) $$E_{i}=\frac{1}{2}v_{i}^{2}+\frac{1}{2}\omega_{i}^{2}x_{i}^{2}+\frac{1}{4}\alpha_{i}x_{i}^{4},\;\;i=1,2,3.$$

The total energy of the continuous system is

(9) $$E_{\mathrm{tot}}=E_{1}+E_{2}+E_{3}+V_{12}+V_{23}+V_{\beta }.$$

Along continuous trajectories, the total-energy balance is

(10) $$\frac{dE_{\mathrm{tot}}}{dt}=-\gamma_{1}v_{1}^{2}-\gamma_{2}v_{2}^{2}-\mu (x_{3}^{2}-1)v_{3}^{2}.$$

Thus, the coupling terms redistribute energy internally without producing a net contribution to the total energy. In the conservative limit, $\gamma_{1}=\gamma_{2}=\mu =0$, with the release mechanism disabled, $\frac{dE_{\mathrm{tot}}}{dt}=0$.

The magnetic-mode energy,

(11) $$E_{3}=\frac{1}{2}v_{3}^{2}+\frac{1}{2}\omega_{3}^{2}x_{3}^{2}+\frac{1}{4}\alpha_{3}x_{3}^{4},$$

is used as the principal energetic observable and defines the threshold for the hybrid release mechanism described in Section 2.3. Because a reset also changes the interaction energies $V_{23}$ and $V_{\beta }$, the magnetic-energy loss $\Delta E_{3}$ and the total system-energy change $\Delta E_{\mathrm{tot}}$ are generally not identical.

The Van der Pol–Duffing representation adopted here is not intended to constitute a unique reduced description of the corresponding solar subsystems. Rather, it provides a minimal phenomenological framework combining amplitude-dependent energy injection or dissipation with nonlinear stiffness. Alternative reduced descriptions could employ Stuart–Landau, generalized Liénard, or other nonlinear oscillators [29,30,31], while inter-mode interactions could be represented by nonlinearities of different order or by state-dependent coupling terms [19,20]. The present formulation was selected because it permits self-sustained dynamics, amplitude regulation, nonlinear frequency shifts, and explicit inter-mode energy exchange within a compact three-mode system. Consequently, the physical conclusions of the present study concern the collective behavior generated by this class of coupled nonlinear energy-exchange systems rather than the uniqueness of the particular polynomial representation.

### 2.3. Hybrid Release and Structural Diagnostic

The continuous dynamics are supplemented by a threshold-triggered magnetic-energy release. An event occurs when

(12) $$E_{3}(t_{k}^{-})=E_{\mathrm{crit}},\;\;{\dot{E}}_{3}(t_{k}^{-})>0.$$

At each event, a fraction η of the magnetic-mode energy is removed,

(13) $$E_{3}(t_{k}^{+})=(1-\eta )E_{3}(t_{k}^{-}).$$

The reset is implemented by scaling the magnetic variables,

(14) $$x_{3}^{+}=q_{k}x_{3}^{-},\;\;v_{3}^{+}=q_{k}v_{3}^{-},$$

where $q_{k}$ is determined numerically so that the prescribed post-release energy is satisfied exactly. Because $E_{3}$ contains the quartic Duffing term, $q_{k}$ is generally not equal to $\sqrt{1-\eta }$; instead, it is obtained numerically from the exact post-release energy condition. The other state variables remain unchanged. Thus, the recurrence time between releases emerges from the subsequent energy recovery rather than being imposed externally.

To characterize continuous magnetic-energy redistribution, we introduce a structural energy-exchange index motivated by the energy-rate interpretation underlying the D-index framework [39]. For continuous-flow segments,

(15) $$I_{\mathrm{str}}^{\mathrm{flow}}=\frac{1}{E_{3}}{\left(\frac{dE_{3}}{dt}\right)}_{\mathrm{flow}}=\frac{-\mu (x_{3}^{2}-1)v_{3}^{2}+k_{23}(x_{2}-x_{3})v_{3}+\beta x_{2}^{2}v_{3}}{E_{3}}.$$

Equivalently,

(16) $$I_{\mathrm{str}}^{\mathrm{flow}}=\frac{d}{dt}\ln E_{3}$$

along continuous trajectory segments. It therefore measures the instantaneous relative rate of magnetic-energy change and is not interpreted as thermodynamic, Shannon, or dynamical entropy.

The diagnostic is evaluated only between release events; discrete changes are characterized separately by the magnetic-energy loss $\Delta E_{3}$ and the total energy jump $\Delta E_{\mathrm{tot}}$ This separation distinguishes continuous energy redistribution from discrete threshold-triggered release.

### 2.4. Model Parameters and Numerical Integration

The baseline parameters are summarized in Table 1. These values characterize the uncoupled modes and do not prescribe the collective period of the full hybrid system. The reference initial condition was

(17) $$y_{0}={\left(0.40,0.10,0.05,0,0,0\right)}^{T}.$$

The baseline parameter set was used as a reference state rather than as a fitted representation of a particular solar cycle. Its purpose was to define a reproducible dynamical regime from which the dependence on initial conditions, intrinsic modal periods, coupling coefficients, magnetic self-excitation, and release parameters could be systematically investigated. These robustness experiments are described in Section 2.7.

Numerical accuracy of both the continuous integration and discrete resets was independently verified as described in Section 2.9.

The baseline numerical values were selected to provide a bounded dynamically active regime in which all three modes participate in the energy exchange and the magnetic subsystem repeatedly reaches the release threshold without entering either threshold-avoidance or unbounded high-energy behavior. They are therefore not treated as uniquely fitted solar parameters. Their purpose is to define a reproducible reference state for the subsequent robustness analysis. The parameter sweeps reported below are essential for distinguishing properties of the proposed mechanism from features specific to this particular numerical choice. In this sense, the present reduced model retains the phenomenological parameterization characteristic of many low-order solar-dynamo models.

The parameters of the reduced model should be interpreted as effective coefficients characterizing different components of the energy dynamics rather than as direct measurements of local solar-plasma properties. The reciprocal coupling coefficients $k_{12}$, $k_{23}$ and β govern energy redistribution among the three explicitly retained collective modes. In particular, $k_{12}$ and $k_{23}$ control the linear exchange between neighboring modes, whereas β determines the nonlinear reciprocal interaction between the tachocline-related and magnetic modes. The coefficients $\gamma_{1}$ and $\gamma_{2}$ represent effective damping of the first two modes, while the Van der Pol coefficient μ controls amplitude-dependent energy injection and dissipation in the magnetic subsystem and therefore strongly affects its recovery rate. The Duffing coefficients $\alpha_{i}$ characterize nonlinear stiffness and the associated amplitude-dependent frequency shifts. Finally, $E_{\mathrm{crit}}$ and η specify the hybrid release mechanism: $E_{\mathrm{crit}}$ sets the magnetic-energy level at which a release is triggered, whereas η determines the fraction of magnetic modal energy removed during each event. Processes not explicitly resolved by the three-mode representation—including additional spatial degrees of freedom, turbulent transport, and other plasma-dynamical effects—are therefore incorporated only implicitly through these effective coefficients. The present parameter values should consequently be regarded as a phenomenological reference set rather than uniquely determined solar quantities.

In principle, some of these effective coefficients could be constrained by observations or higher-dimensional simulations. The intrinsic frequencies may be related to characteristic activity timescales, while the coupling and nonlinear coefficients could be estimated by requiring the reduced model to reproduce observed spectral properties, phase relationships, recovery times, and statistical characteristics of magnetic-energy proxies. Alternatively, reduced coefficients could be inferred from global MHD simulations by projecting the simulated dynamics onto a small set of collective modes. Such an inverse calibration is beyond the scope of the present work, whose purpose is to test the dynamical mechanism and its robustness rather than to obtain a uniquely fitted solar parameter set.

For the numerical implementation, the continuous dynamics were integrated using the DOP853 adaptive explicit Runge–Kutta method with relative and absolute tolerances $rtol=10^{-10}$ and $atol=10^{-12}$, respectively, and a maximum internal step of 0.05 yr. The baseline simulation covered 1200 yr, with the first 150 yr excluded as a transient. The trajectory was stored on a uniform output grid with $\Delta t_{\mathrm{out}}$ = 0.05 yr. The uniform output interval is distinct from the adaptive internal integration steps and therefore does not determine either the integration accuracy or the event times. Pre- and post-event states were recorded separately for event and energy diagnostics.

The hybrid system was integrated piecewise between successive threshold crossings. The release condition was defined by the event function

(18) $$h(y)=E_{3}(y)-E_{\mathrm{crit}},$$

with only upward crossings accepted. Each event terminated the current continuous integration segment, and the solver-localized event state was taken as the pre-release state $y^{-}$. The nonlinear reset factor $q_{k}$ was then determined using Brent’s method from

(19) $$E_{3}(q_{k}x_{3}^{-},q_{k}v_{3}^{-})=(1-\eta )E_{3}(x_{3}^{-},v_{3}^{-}),$$

with root-finding tolerances $xtol=10^{-14}$ and $rtol=10^{-13}$. After application of the reset, integration was restarted at the same physical time from the post-release state $y^{+}$. Because $E_{3}^{+}<E_{\mathrm{crit}}$, an immediate repeated upward crossing cannot occur.

### 2.5. Spectral and Event-Based Diagnostics

The long-term dynamics were characterized using two complementary timescales: the dominant spectral period of the magnetic activity and the recurrence time of energy-release events. The magnetic activity observable was defined as $A(t)=|x_{3}(t)|$.

After removal of the transient and temporal mean, its dominant period was estimated independently using FFT and Welch power spectra,

(20) $$T_{\mathrm{FFT}}=\frac{1}{f_{\mathrm{FFT}}^{\max}},\;\;T_{\mathrm{Welch}}=\frac{1}{f_{\mathrm{Welch}}^{\max}}.$$

Prior to spectral estimation, the analyzed signal was linearly detrended. For the Welch estimate, a Hann window with $N_{\mathrm{seg}}=\min(4096,N)$, 50% overlap, linear segment detrending, and density scaling was used. For the baseline output interval $\Delta t_{\mathrm{out}}$ = 0.05 yr, $N_{\mathrm{seg}}$ = 4096 corresponds to a 204.8 yr segment duration. The dominant spectral peak was searched over periods of 1–40 yr and refined by parabolic interpolation of the logarithmic spectral power around the discrete maximum. Time-resolved spectra were evaluated in 250 yr windows advanced in 100 yr increments.

No prescribed cycle length or minimum peak-separation criterion was used. Time-resolved spectral analysis was additionally applied to assess the persistence of the dominant timescale throughout the simulation.

Release times $t_{k}$ were determined directly from the threshold crossings defined in Section 2.3, and the waiting intervals were calculated as $\Delta t_{k}=t_{k}-t_{k-1}$.

Their organization was characterized by the mean, median, standard deviation, coefficient of variation, and release rate. Bootstrap confidence intervals were used to assess the uncertainty of the mean waiting time.

The spectral period and mean release interval were treated as independent diagnostics. Their relationship was quantified by

(21) $$Q=\frac{\langle \Delta t\rangle }{T_{\mathrm{Welch}}}$$

Values Q≈1 indicate approximately one release per dominant cycle, whereas Q>1 identifies regimes in which one or more oscillatory excursions fail to reach $E_{\mathrm{crit}}$, producing event skipping. This combined analysis therefore distinguishes the continuous oscillatory timescale from the recurrence timescale of discrete energy-release events.

The sensitivity of spectral and peak-based cycle estimates to smoothing and peak-detection parameters was evaluated using the unchanged baseline trajectory, while the effect of independent variations in the physical release threshold was assessed through separate integrations (Appendix E).

### 2.6. Phase-Space and Event-Based Dynamical Analysis

The organization of the hybrid dynamics was examined using phase-space and event-conditioned diagnostics. Continuous magnetic dynamics were visualized in the $\left(x_{3},v_{3}\right)$ plane, while the pre-release states $\left(x_{2}\left(t_{k}^{-}\right),v_{2}\left(t_{k}^{-}\right)\right)$ were used to characterize the tachocline-related mode at threshold crossings.

The tachocline phase at each release was defined as

(22) $$\phi_{2,k}=\mathrm{atan}2\left(-\frac{v_{2}\left(t_{k}^{-}\right)}{\omega_{2}},x_{2}\left(t_{k}^{-}\right)\right)\;\left(\mathrm{mod}\;2\pi \right).$$

Here, atan2(y,x) denotes the two-argument, quadrant-aware extension of the standard inverse tangent to the full circle, with the first argument corresponding to the y-coordinate and the second to the x-coordinate. Thus, in Equation (22), $y=-v_{2}/\omega_{2}$ and $x=x_{2}$; away from the coordinate axes, the corresponding tangent ratio is $y/x=(-v_{2}/\omega_{2})/x_{2})$, while atan2 preserves the correct quadrant.

Phase concentration was quantified by the circular mean and mean resultant length

(23) $$R=\left|\frac{1}{N}\sum_{k=1}^{N}e^{i\phi_{2,k}}\right|,$$

with departure from phase uniformity assessed using the Rayleigh test. Significant concentration was interpreted as a phase preference of release events rather than strict phase locking.

Event-to-event organization was examined using the recurrence map $\left(\Delta t_{k},\Delta t_{k+1}\right),$ and local cycle durations were compared with the corresponding release intervals using Pearson and Spearman correlations. These diagnostics distinguish global agreement of characteristic timescales from direct cycle-to-cycle synchronization.

Because phase-space and recurrence diagnostics alone cannot establish quasiperiodicity or exclude weak chaotic instability, dynamical stability was evaluated independently using the hybrid Lyapunov analysis described in Section 2.8.

### 2.7. Robustness Experiments

Robustness was assessed with respect to initial conditions, intrinsic modal periods, hybrid-release parameters, and the principal continuous-system parameters. An ensemble of *n* = 30 simulations with ±5% perturbations of the baseline initial state was used to test trajectory sensitivity.

To determine whether the collective timescale is inherited from the prescribed modal periods, independent sweeps were performed over $T_{1}$ = 16–28 yr, $T_{2}$ = 8–14 yr, and $T_{3}$ = 3.5–6.5 yr supplemented by a joint N=24 ensemble with simultaneous ±20% variations in all three periods. A finer $T_{2}$ sweep and a two-dimensional $T_{2},\eta$ map were used to resolve localized event-skipping regimes.

Sensitivity to the release mechanism was examined by varying η and $E_{\mathrm{crit}}$, including the $E_{\mathrm{crit}}-\eta$ parameter plane. The parameters $k_{12},\;k_{23},\;\mu ,\;\alpha_{3}$ were independently varied over $0.7p_{0}\leq p\leq 1.3p_{0}$, where $p_{0}$ is the baseline value.

For each experiment, $T_{Welch}$, the mean release interval, the release rate, and the occurrence of event skipping were evaluated. Robustness was defined as persistence of the qualitative hybrid regime within the investigated parameter neighborhood, rather than invariance of its characteristic timescale.

### 2.8. Hybrid Lyapunov Analysis

Dynamical stability was evaluated using a Lyapunov analysis formulated for the hybrid system. Between release events, infinitesimal perturbations obey the variational equations

(24) $$\delta \overset{\cdot }{y}=J\left(y\right)\delta y,$$

where J=∂F/∂y. At each threshold-triggered reset, perturbations were propagated using the saltation matrix.

Because the reset factor $q_{k}$ is determined from the exact magnetic-energy condition, it is generally state dependent. The corresponding dependence was therefore retained explicitly in the reset Jacobian. Let

(25) $$z={\left(x_{3},v_{3}\right)}^{T},\;\;c=1-\eta ,$$

and define

(26) $$G(q,z)=E_{3}(qz)-cE_{3}(z)=0.$$

Implicit differentiation gives

(27) $$\frac{\partial q}{\partial z_{j}}=-\frac{q{\left[\nabla E_{3}(qz)\right]}_{j}-c{\left[\nabla E_{3}(z)\right]}_{j}}{\nabla E_{3}(qz)\cdot z}.$$

Accordingly, the magnetic block of the reset Jacobian is

(28) $$DR_{z}=qI+z{\left(\nabla q\right)}^{T}=\left(\begin{array}{cc} q+x_{3}q_{x_{3}} & x_{3}q_{v_{3}}\\ v_{3}q_{x_{3}} & q+v_{3}q_{v_{3}} \end{array}\right).$$

For the state ordering (Equation (1)) the full 6×6 reset Jacobian is the identity except for this $\left(x_{3},v_{3}\right)$ block.

For event function by Equation (18), the event-surface normal is

(29) $$n=\nabla h={\left(0,0,\omega_{3}^{2}x_{3}+\alpha_{3}x_{3}^{3},0,0,v_{3}\right)}^{T}.$$

The tangent perturbations were transformed at every threshold crossing using the complete saltation matrix

(30) $$S=DR+\frac{\left(f^{+}-DRf^{-}\right)n^{T}}{n^{T}f^{-}},$$

Here,

(31) $$f^{-}=F(y^{-}),\;\;f^{+}=F(y^{+}),$$

denote the continuous vector fields evaluated immediately before and after the reset, respectively. The denominator of the saltation term represents the rate at which the pre-event trajectory crosses the event surface:

(32) $$n^{T}f^{-}=\nabla h{\left(y^{-}\right)}^{T}F(y^{-})={\left.\frac{dh}{dt}\right|}_{t_{k}^{-}}={\dot{E}}_{3}(t_{k}^{-}).$$

Because only upward threshold crossings are accepted, ${\dot{E}}_{3}(t_{k}^{-})>0$ for the release events considered here, so the denominator is nonzero for transverse crossings. Near-grazing events correspond to $n^{T}f^{-}\to 0$ and were monitored separately; none occurred in the reported calculations.

The analytical state-dependent reset Jacobian was independently verified against a central finite-difference approximation of the complete nonlinear reset map at 20 actual threshold crossings. The median and maximum best relative Frobenius discrepancies were $6.73\times 10^{-13}$ and $1.20\times 10^{-12}$, respectively. The maximum relative error in satisfying the prescribed post-reset magnetic-energy condition was $4.13\times 10^{-16}$. Details of the validation and its finite-difference step-size dependence are provided in Appendix F (Figure A12).

The Lyapunov spectrum was estimated by periodic QR orthonormalization,

(33) $$\lambda_{i}\left(T\right)=\frac{1}{T}\underset{j}{\sum }\ln\left|{\left(R_{j}\right)}_{ii}\right|.$$

The state and tangent equations were propagated as a common augmented system using the same adaptive DOP853 integration procedure. For the final extended Lyapunov calculations, $rtol=2\times 10^{-10}$, $atol=2\times 10^{-12}$, and a maximum internal step of 0.05 yr were used. QR orthonormalization was performed at intervals of 0.5 yr. Numerical sensitivity was evaluated independently with respect to the accumulation horizon, QR interval, and solver tolerances. No fixed numerical tolerance was subsequently used to classify an exponent as zero, positive, or negative.

Robustness of $\lambda_{max}$ was additionally tested by varying $k_{12},\;k_{23},\;\mu ,\;\alpha_{3},\;\eta ,\;E_{\mathrm{crit}}$. The sign of a small finite-time exponent was not used alone to classify the dynamics; interpretation was based on its long-time convergence and parameter robustness. In particular, a near-zero $\lambda_{max}$ was not regarded as sufficient evidence of quasiperiodicity.

### 2.9. Numerical Validation

Numerical consistency was verified independently for the continuous dynamics and the discrete reset mechanism. In the conservative limit, $\gamma_{1}=\gamma_{2}=\mu =0,$ with hybrid releases disabled, the analytical energy balance requires $\frac{dE_{\mathrm{tot}}}{dt}=0$.

The maximum relative numerical energy deviation,

(34) $$\varepsilon_{E}^{\max}=\underset{t}{\max}\frac{\left|E_{\mathrm{tot}}\left(t\right)-E_{\mathrm{tot}}\left(0\right)\right|}{\left|E_{\mathrm{tot}}\left(0\right)\right|},$$

was $5.67\times 10^{-15}$, confirming the consistency of the continuous equations and reciprocal coupling formulation.

The reset implementation was validated from

(35) $$\varepsilon_{\mathrm{reset},k}=\frac{\left|E_{3,k}^{+}-\left(1-\eta \right)E_{3,k}^{-}\right|}{\left(1-\eta \right)E_{3,k}^{-}}.$$

The maximum relative reset error was $4.13\times 10^{-16}$, showing that the prescribed post-release energy was reproduced to numerical precision. These tests independently validate the continuous and discrete components of the hybrid numerical scheme.

## 3. Results

### 3.1. Emergence of a Persistent Hybrid Energy-Release Regime

**Figure 1:**
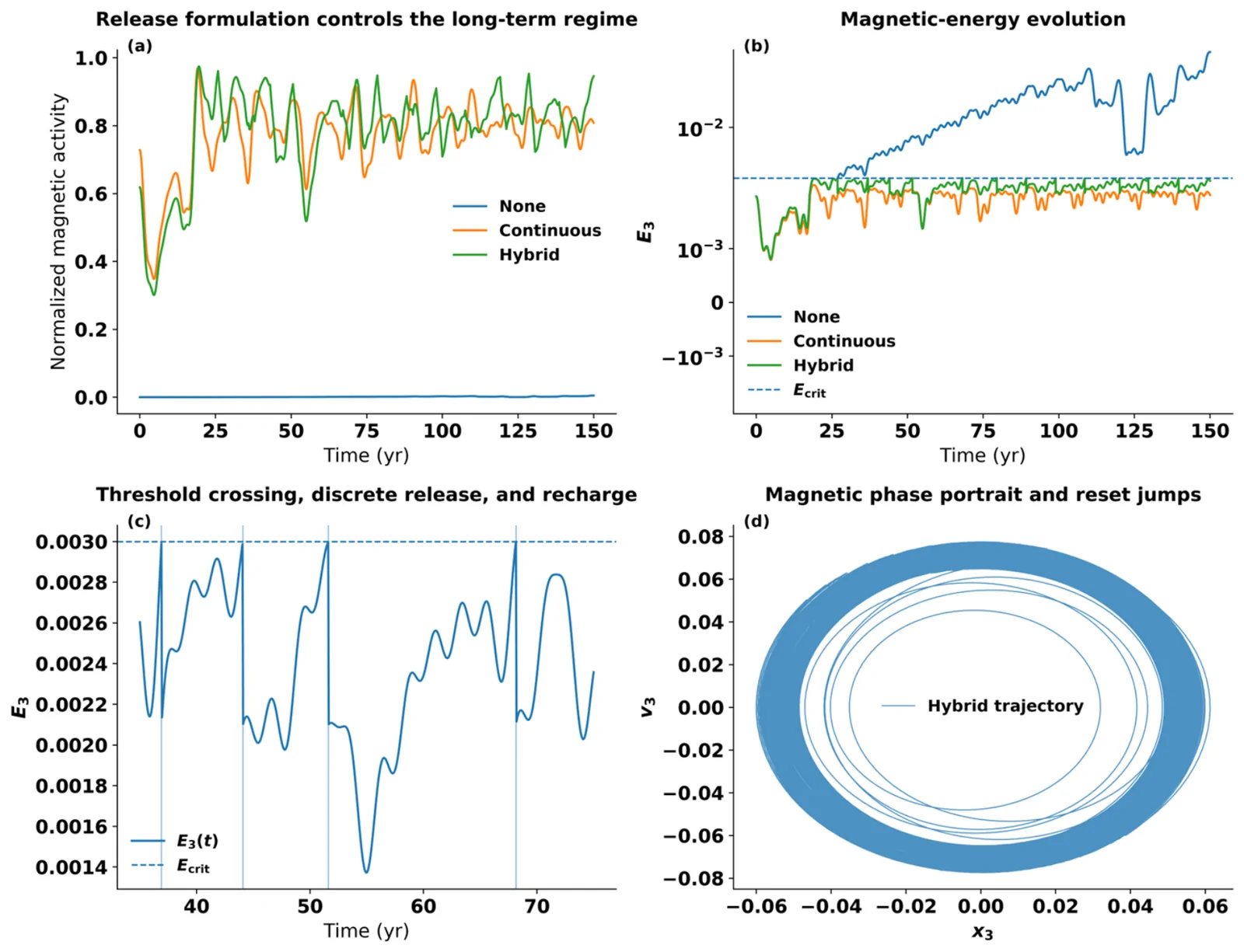
Emergence of the hybrid accumulation–release regime. (**a**) Normalized magnetic activity for the no-release, continuous-loss, and hybrid formulations. (**b**) Magnetic energy $E_{3}$ relative to the critical threshold $E_{\mathrm{crit}}$. (**c**) Hybrid accumulation, threshold crossing, discrete energy release, and subsequent recharge. (**d**) Magnetic phase-space trajectory ($x_{3}$, $v_{3}$) illustrating continuous evolution interrupted by threshold-triggered resets.

The long-term behavior of the three-mode system depends strongly on the representation of magnetic-energy release. To isolate the dynamical role of the release mechanism, three otherwise identical formulations were compared: (i) a no-release system, in which the continuous dynamics evolve without an explicit energy-removal mechanism; (ii) a continuous-loss formulation, in which magnetic dissipation increases smoothly near the prescribed energy threshold; and (iii) the hybrid formulation introduced here, in which the continuous evolution is interrupted by discrete threshold-triggered release events. The corresponding trajectories are compared in Figure 1.

The three formulations exhibit qualitatively different energetic behavior. In the no-release case, the magnetic subsystem is not constrained by a threshold-reset mechanism. Although the magnetic-activity observable remains oscillatory over the displayed interval (Figure 1a), the corresponding modal energy $E_{3}$ exceeds the reference threshold $E_{\mathrm{crit}}$ and evolves toward a substantially higher-energy state (Figure 1b). Thus, removal of the release mechanism changes the long-term energetic regime rather than merely eliminating identifiable release events.

In contrast, the hybrid formulation produces a bounded accumulation–release regime. Magnetic energy repeatedly approaches $E_{\mathrm{crit}}=3.0\times 10^{-3}$, and each upward crossing triggers a discrete reset (Figure 1c). For the baseline release depth η = 0.30, the prescribed post-release magnetic energy is $E_{3}^{+}=2.1\times 10^{-3}$, corresponding to an intrinsic magnetic-energy reduction $\Delta E_{3}=9.0\times 10^{-4}$.

The subsystem then recovers through the autonomous continuous dynamics until the next threshold crossing. The phase portrait in Figure 1d illustrates the resulting hybrid motion: bounded continuous evolution is interrupted by discrete reset-induced jumps.

Over the 1200 yr baseline simulation, 116 release events are obtained, including 104 after removal of the initial 150 yr transient. The post-transient dynamics exhibit a dominant approximately decadal timescale, $T_{\mathrm{FFT}}$ = 10.084 yr, $T_{\mathrm{Welch}}$ = 10.085 yr.

Thus, the threshold-triggered reset is an active component of the long-term dynamics rather than a passive event-detection rule. The hybrid model establishes a persistent bounded sequence of energy accumulation, release, and recovery, whereas removing or smoothing the release mechanism produces qualitatively different regimes. Whether the resulting decadal timescale is tied to a prescribed modal period or instead represents a collective property of the coupled hybrid dynamics is examined systematically in Section 3.3, Section 3.4, Section 3.5, Section 3.6 and Section 3.7.

### 3.2. Long-Term Persistence of the Decadal Collective Timescale

**Figure 2:**
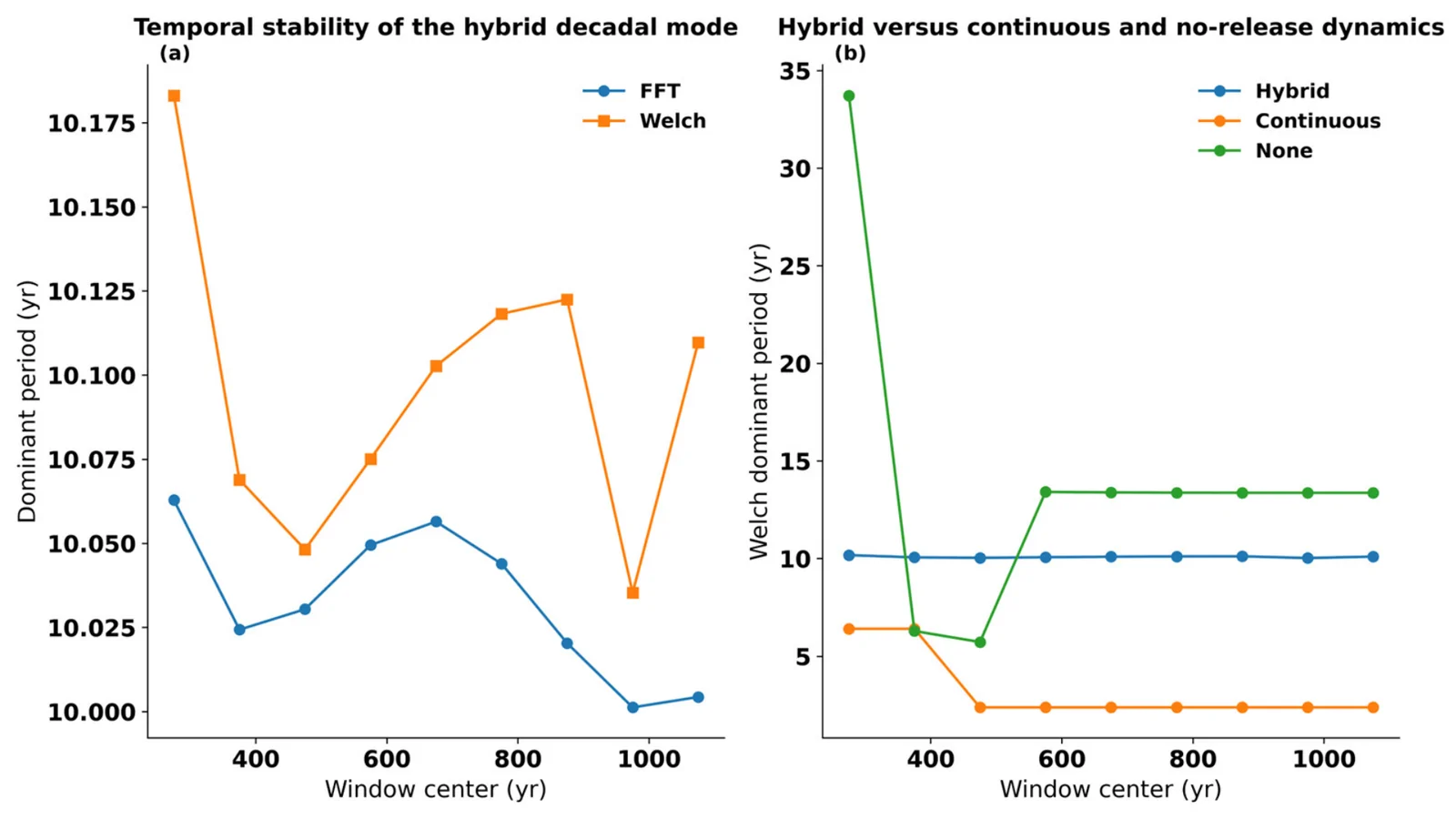
Long-term spectral stability of the hybrid regime. (**a**) Time-resolved FFT and Welch dominant periods of the hybrid model over the post-transient evolution. (**b**) Welch-period evolution for the hybrid, continuous-loss, and no-release formulations.

As shown in Figure 2a, the dominant FFT and Welch periods of the hybrid model remain tightly clustered around 10 yr throughout the analyzed interval. The FFT estimates vary only slightly around 10.0–10.1 yr, while the Welch estimates remain approximately within 10.0–10.2 yr. No systematic long-term drift toward either shorter or longer periods is observed.

This behavior contrasts with the continuous-loss and no-release formulations (Figure 2b). The continuous-loss model evolves from an initial period of approximately 6.4 yr toward a much shorter long-term period of about 2.4 yr. The no-release system exhibits an even stronger transient evolution, with the dominant period initially exceeding 30 yr before approaching approximately 13.5 yr at later times. Thus, neither alternative formulation maintains the persistent decadal organization observed in the hybrid model.

For the complete post-transient baseline trajectory, the independently determined spectral estimates are $T_{\mathrm{FFT}}$ = 10.084 yr, $T_{\mathrm{Welch}}$ = 10.085 yr.

The close agreement between the two estimators and their stability across successive time windows indicate that the approximately decadal timescale is a persistent property of the established hybrid regime rather than a transient spectral feature. Additional post-processing tests further show that this spectral estimate is essentially insensitive to smoothing and peak-detection choices (Appendix E).

These results establish the temporal stability of the collective timescale. Whether this timescale is inherited from the prescribed intrinsic modal periods or instead emerges from the coupled accumulation–release dynamics is examined through the robustness experiments below.

### 3.3. Robustness to Initial Conditions

To determine whether the persistent decadal regime depends on a particular choice of the initial state, an ensemble of 30 simulations was generated by independently perturbing the baseline initial conditions by up to 5%. All model parameters were kept fixed, and each realization was integrated for 700 yr, with the first 150 yr excluded from the analysis. For every realization, the dominant Welch period, release rate, and waiting-time statistics were evaluated using the same procedure as for the baseline simulation.

**Figure 3:**
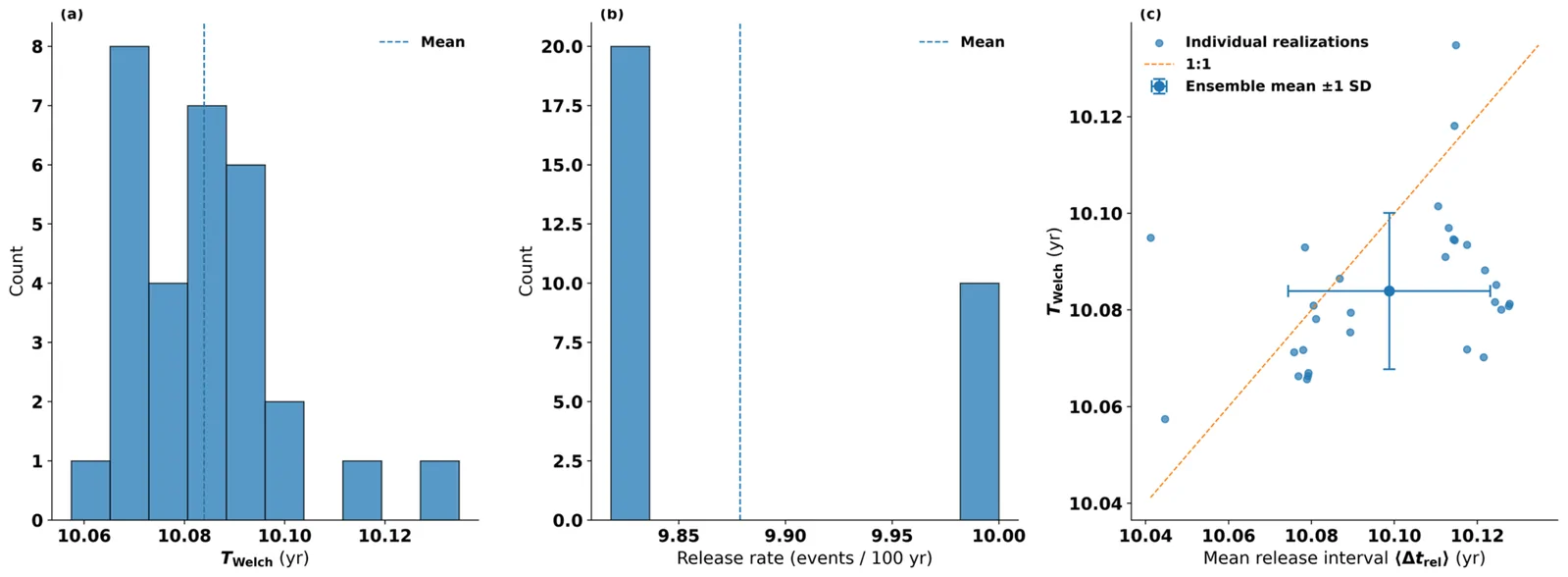
Robustness of the hybrid regime to initial conditions. (**a**) Distribution of the dominant Welch period $T_{\mathrm{Welch}}$ across N = 30 perturbed initial conditions; the dashed line indicates the ensemble mean. (**b**) Distribution of the post-transient energy-release rate; the dashed line denotes the ensemble mean. (**c**) Relationship between the mean release interval $\langle \Delta t_{\mathrm{rel}}\rangle$ and the dominant Welch period for individual realizations. The dashed line indicates $T_{\mathrm{Welch}}=\langle \Delta t_{\mathrm{rel}}\rangle$. The enlarged marker with horizontal and vertical error bars denotes the ensemble mean ±1 standard deviation of $\langle \Delta t_{\mathrm{rel}}\rangle$ and $T_{\mathrm{Welch}}$, respectively.

Despite the resulting differences in individual trajectories, the dominant collective timescale remained tightly concentrated (Figure 3a). The ensemble-mean Welch period was $\langle T_{\mathrm{Welch}}\rangle$ = 10.0839 yr, with a standard deviation of only $\sigma_{T}$ = 0.0162 yr, and the individual estimates ranged from 10.057 to 10.135 yr. Thus, the dispersion of the collective period is small compared with its approximately decadal value.

The energy-release recurrence was similarly stable (Figure 3b). Individual realizations contained either 54 or 55 post-transient release events, corresponding to release rates of 9.82–10.00 events per 100 yr. The ensemble-average rate was $\langle R_{\mathrm{rel}}\rangle$ = 9.88 events/100 yr, while the mean release interval was $\langle \Delta t_{\mathrm{rel}}\rangle$ = 10.099 yr.

The coefficient of variation of the release intervals remained low, with an ensemble mean of approximately 0.102, indicating relatively regular recurrence while allowing finite event-to-event variability.

The relation between the spectral and release timescales is shown in Figure 3c. Most realizations remain close to the 1:1 reference line, $T_{\mathrm{Welch}}\approx \langle \Delta t_{\mathrm{rel}}\rangle ,$ demonstrating that the decadal spectral organization and the characteristic recharge–release recurrence persist simultaneously under perturbations of the initial state. The small deviations from the 1:1 relation reflect local variability rather than a transition to a different dynamical regime.

Overall, the initial-condition ensemble demonstrates that the persistent hybrid regime and its approximately decadal collective timescale are not specific to a single baseline trajectory. This result complements the long-time spectral analysis of Section 3.2 and motivates the subsequent examination of whether the same collective timescale remains robust when the parameters governing the accumulation–release dynamics and the intrinsic modal periods are varied.

### 3.4. Release Depth Controls the Collective Timescale

**Figure 4:**
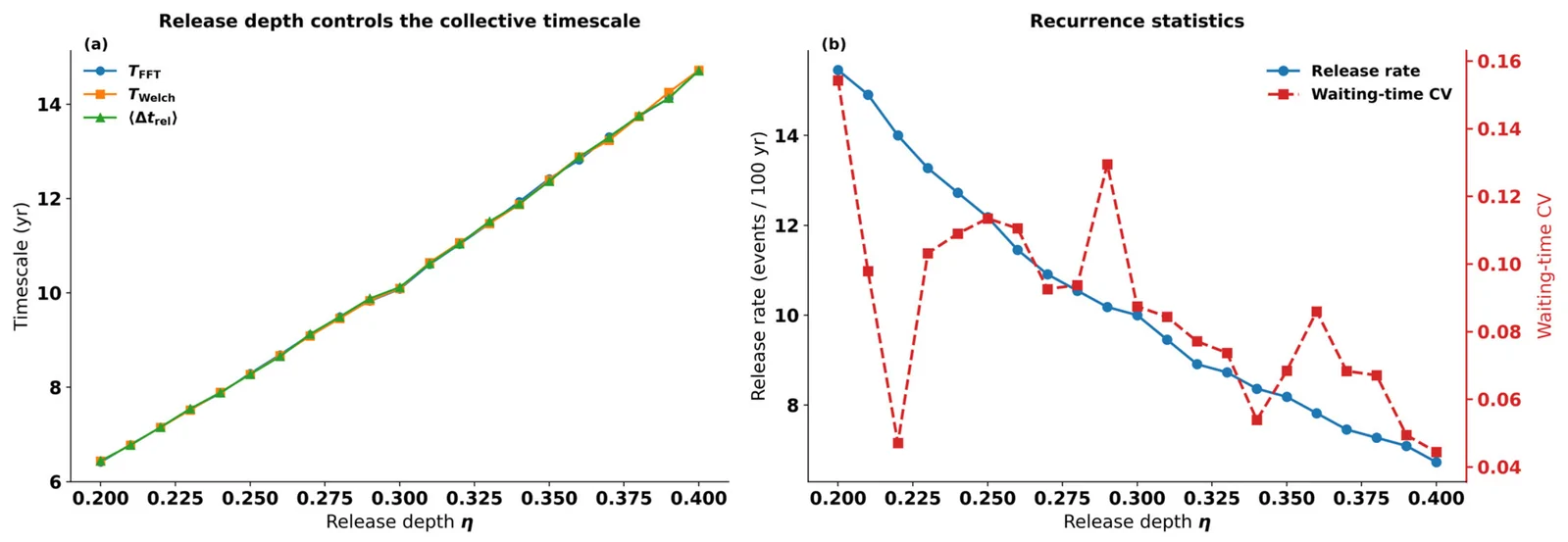
Dependence of the collective timescale on release depth. (**a**) FFT period $T_{\mathrm{FFT}}$, Welch period $T_{\mathrm{Welch}}$, and mean release interval $\langle \Delta t_{\mathrm{rel}}\rangle$ as functions of the release depth η. (**b**) Release rate and coefficient of variation (CV) of the waiting times as functions of η.

To quantify the role of the hybrid release mechanism in setting the characteristic timescale, the fractional release depth η was varied while all other parameters were kept at their baseline values. The release depth η has a pronounced effect on the characteristic timescale of the hybrid regime. As shown in Figure 4a, increasing η produces a nearly monotonic increase in both the dominant spectral period and the mean interval between successive release events. Over the investigated range 0.20≤η≤0.40, the Welch period increases from approximately $T_{\mathrm{Welch}}$ = 6.44 yr at η = 0.20 to $T_{\mathrm{Welch}}$ = 14.72 yr at η = 0.40. The baseline value η = 0.30 gives $T_{\mathrm{Welch}}≃10.09$ yr.

The close agreement among $T_{\mathrm{FFT}}$, $T_{\mathrm{Welch}}$, and the mean release interval $\langle \Delta t_{\mathrm{rel}}\rangle$ throughout this sweep (Figure 4a) shows that the dominant spectral timescale follows the accumulation–release recurrence. In the approximately decadal range, corresponding roughly to 0.27≲η≲0.34, all three estimates remain closely aligned.

This dependence has a direct interpretation within the hybrid formulation. At each threshold crossing, $E_{3}^{+}=(1-\eta )E_{\mathrm{crit}}$, so increasing η produces a larger post-release energy deficit, $\Delta E_{3}=\eta E_{\mathrm{crit}}$.

A deeper release therefore requires a longer recovery before $E_{3}$ reaches the threshold again. The collective period consequently reflects the recovery dynamics of the coupled system rather than a fixed externally prescribed cycle.

The recurrence statistics support this interpretation (Figure 4b). As η increases, the release rate decreases systematically from approximately 15.5 to 6.8 events per 100 yr, consistent with the corresponding increase in the mean waiting time. The waiting-time coefficient of variation remains relatively small, approximately 0.04–0.15, indicating that the change in timescale occurs within a comparatively regular release regime rather than through strongly irregular recurrence.

This result provides direct evidence that the decadal timescale is dynamically controlled by the hybrid accumulation–release process. The extent to which it also depends on the prescribed intrinsic modal periods is examined in Section 3.5.

### 3.5. Robustness to the Prescribed Intrinsic Modal Periods

**Figure 5:**
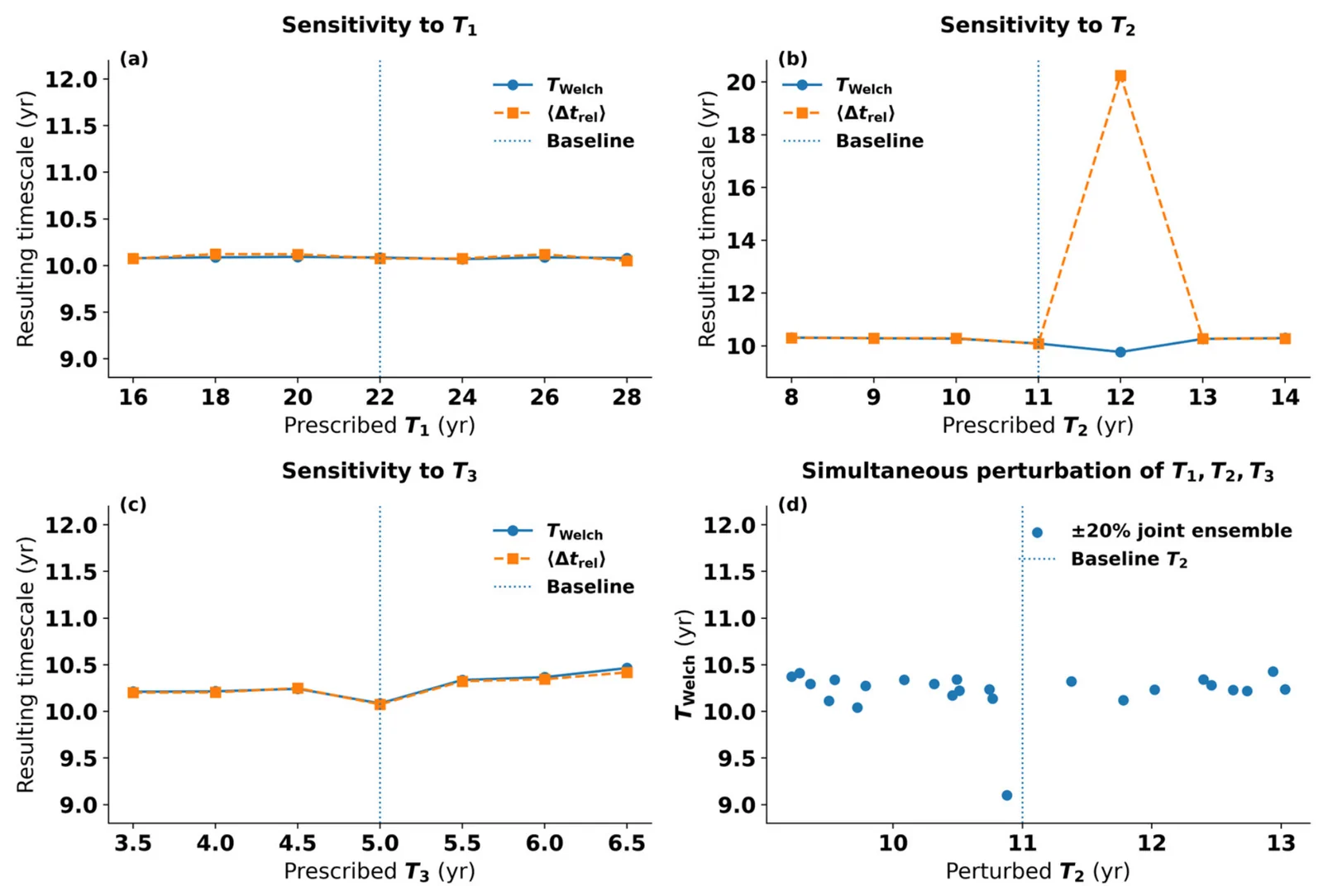
Robustness of the collective timescale to the intrinsic modal periods. Dependence of the dominant Welch period on (**a**) $T_{1}$, (**b**) $T_{2}$, and (**c**) $T_{3}$, with the remaining parameters fixed. (**d**) Distribution obtained when all three periods are simultaneously varied within ±20% of their baseline values.

A central question is whether the approximately decadal collective timescale is simply inherited from one of the prescribed intrinsic modal periods. This was tested by independently varying $T_{1}$, $T_{2}$, and $T_{3}$, followed by a joint perturbation of all three periods (Figure 5).

Variation of the slow-mode period over $T_{1}$ = 16–28 yr has almost no effect on the resulting dynamics (Figure 5a). Both $T_{\mathrm{Welch}}$ and the mean release interval remain close to 10.1 yr throughout the tested range. Thus, the collective period does not appreciably scale with the prescribed slow-mode timescale over the investigated range.

The tachocline-related period $T_{2}$ was varied from 8 to 14 yr (Figure 5b). Despite this substantial change, $T_{\mathrm{Welch}}$ remains close to 10 yr across the sweep, including prescribing $T_{2}$ = 8 yr which produces $T_{\mathrm{Welch}}≃10.31\mathrm{yr}$, and $T_{2}$ = 14 yr gives $T_{\mathrm{Welch}}≃10.29\mathrm{yr}$.

A localized exception occurs near $T_{2}$ = 12 yr, where the mean release interval increases to approximately 20 yr while the spectral period remains near 9.8 yr. This separation indicates event skipping rather than direct entrainment of the collective period by $T_{2}$; the transition is examined in greater detail in Section 3.6.

Variation of the magnetic-mode period over $T_{3}$ = 3.5–6.5 yr also leaves the resulting collective period within a narrow decadal range (Figure 5c), although a modest systematic dependence is visible at larger $T_{3}$. The close correspondence between $T_{\mathrm{Welch}}$ and $\langle \Delta t_{\mathrm{rel}}\rangle$ is retained throughout this one-parameter sweep.

Finally, all three intrinsic periods were varied simultaneously by up to ±20% from their baseline values (Figure 5d). Across the N=24 joint realizations, $\langle T_{\mathrm{Welch}}\rangle$ = 10.213 yr, with a standard deviation of approximately 0.256 yr. All 24 realizations remained within the 9–12 yr range, and 23 of 24 remained between 9.5 and 11 yr.

These results demonstrate that the decadal collective timescale is not a direct copy of the prescribed $T_{2}$ = 11 yr period, nor does it track $T_{1}$ or $T_{3}$ individually. Rather, it persists under substantial independent and simultaneous variations in the intrinsic modal periods, supporting its interpretation as an emergent property of the coupled hybrid dynamics.

### 3.6. Parameter-Dependent Dynamical Regimes and Event Skipping

**Figure 6:**
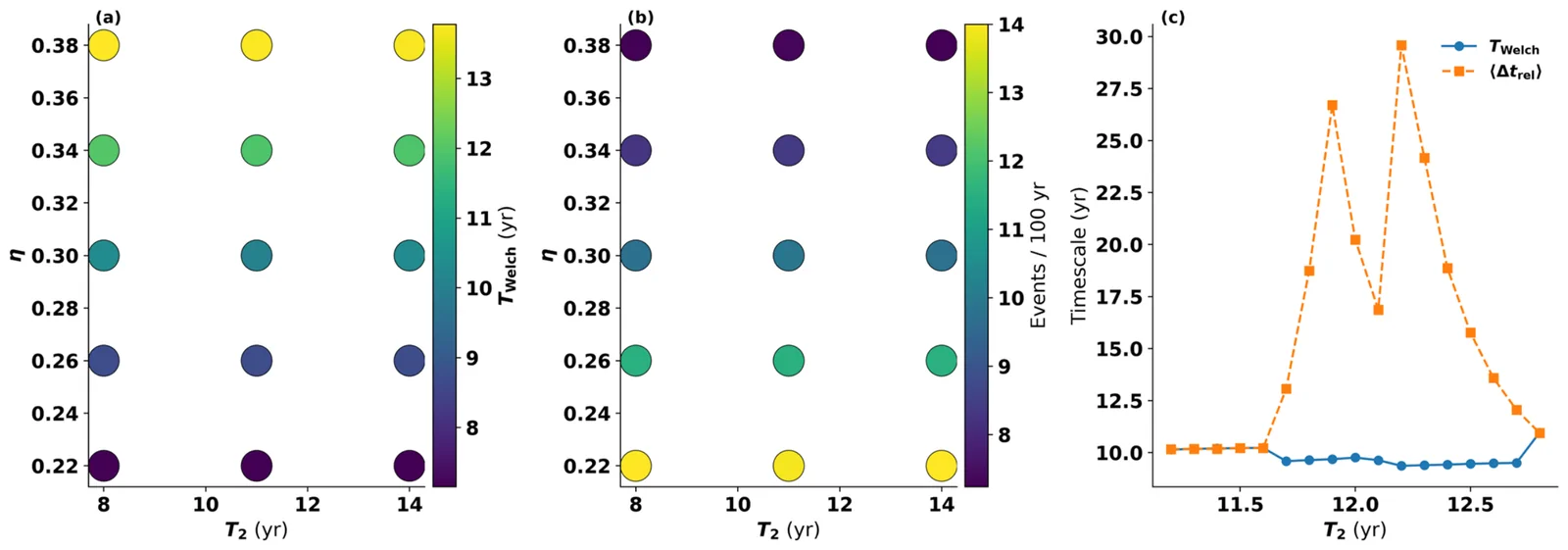
Parameter-dependent regimes and event skipping. (**a**) Dominant collective period $T_{Welch}$ and (**b**) energy-release rate obtained for the investigated combinations of the prescribed tachocline-related period $T_{2}$ and release depth η. Each colored circle represents an explicitly simulated parameter combination, with marker color indicating the corresponding calculated value. (**c**) Fine scan in $T_{2}$, showing the dominant Welch period and mean release interval across the localized event-skipping transition.

The combined influence of the tachocline-related intrinsic period $T_{2}$ and the release depth η was examined to determine how the collective spectral timescale and release recurrence vary across the parameter space. The resulting $T_{2}-\eta$ map reveals that the long-term dynamics are controlled much more strongly by the release depth than by $T_{2}$ over the investigated ranges (Figure 6a,b).

As shown in Figure 6a, the dominant Welch period changes systematically with η, increasing from approximately 7 yr at small release depths to more than 13 yr at the largest values considered. In contrast, changing $T_{2}$ from 8 to 14 yr produces only comparatively small variations at fixed η. The approximately decadal region occurs near the baseline release depth, with the $T_{\mathrm{Welch}}$ = 10 yr contour lying close to η≃0.30. The corresponding release rate decreases systematically with increasing η (Figure 6b), consistent with the longer recovery time required after deeper releases.

Although this behavior is regular over most of the parameter domain, a finer scan of $T_{2}$ reveals a localized transition in the organization of release events (Figure 6c). For $T_{2}≲11.6$ yr, the spectral period and mean release interval are both approximately 10.2 yr. Between approximately $T_{2}$ = 11.7 and 12.7 yr, the two timescales separate markedly. The Welch period remains close to $T_{\mathrm{Welch}}\approx 9.4-9.8$ yr, whereas the mean release interval increases intermittently to approximately 13–30 yr.

This separation indicates event skipping: the underlying oscillatory dynamics continue on an approximately decadal timescale, but some excursions fail to reach the release threshold. Consequently, a threshold-triggered release does not occur during every oscillatory cycle, and $\langle \Delta t_{\mathrm{rel}}\rangle >T_{\mathrm{Welch}}$.

At $T_{2}≃12.8$ yr, the spectral and release timescales become closely aligned again at approximately 11 yr (Figure 6c), indicating recovery of a more regular one-release-per-cycle regime.

These results demonstrate that agreement between the spectral and release timescales is characteristic of the regular hybrid regime but is not a universal identity. More generally, $T_{\mathrm{collective}}\neq \langle \Delta t_{\mathrm{rel}}\rangle$ when threshold crossings are skipped. The $T_{2}-\eta$ analysis therefore supports two complementary conclusions: the collective period is controlled predominantly by the accumulation–release dynamics over the tested parameter range, while the intrinsic modal dynamics can locally reorganize the timing of threshold crossings and produce event-skipping regimes.

### 3.7. Robustness to Coupling and Magnetic-Mode Parameters

The robustness analysis was extended beyond the intrinsic modal periods by varying the parameters that control intermode coupling and the nonlinear magnetic dynamics. Specifically,$k_{12}$, $k_{23}$, μ, and $\alpha_{3}$ were independently varied between 0.70 and 1.3 times their baseline values, while all other parameters were held fixed. The corresponding dominant Welch periods and mean release intervals are summarized in Appendix B (Figure A3).

The collective timescale is only weakly affected by variations in the coupling coefficients. Across the tested range of $k_{12}$, the dominant period remains between approximately 10.08 and 10.19 yr. A similarly narrow interval is obtained for $k_{23}$. Thus, moderate changes in the strength of coupling between the three effective modes do not substantially alter the decadal regime.

The magnetic Duffing coefficient $\alpha_{3}$ has an even weaker influence. Its variation over the same relative range changes the dominant period by less than 0.01 yr. The persistence of the characteristic timescale under these variations indicates that the decadal behavior is not critically dependent on a narrowly selected nonlinear stiffness of the magnetic mode.

A markedly different dependence is found for the magnetic self-excitation parameter μ. Increasing μ systematically decreases the dominant period, from approximately $T_{\mathrm{Welch}}≃14.69\;\mathrm{yr}$ at 0.7 $\mu_{0}$ to $T_{\mathrm{Welch}}≃7.88\;\mathrm{yr}$ at 1.3$\mu_{0}$. The mean interval between successive release events follows nearly the same dependence. Hence, μ acts as a strong control parameter of the accumulation–release timescale.

This result provides a physical complement to the dependence on the release depth η established in Section 3.4. The two parameters influence different stages of the hybrid cycle. The release depth determines the post-event magnetic-energy deficit, $\Delta E_{\mathrm{recharge}}=\eta E_{\mathrm{crit}}$, whereas μ controls the self-excitation of the magnetic subsystem and thereby influences the rate at which this deficit is recovered.

The parameter analysis thus reveals a clear hierarchy of sensitivities. The collective decadal regime is robust to moderate variations in $k_{12}$, $k_{23}$, and $\alpha_{3}$, whereas μ, together with the release depth η, provides an effective control of the magnetic recharge timescale. This demonstrates that the observed robustness does not result from general parameter insensitivity, but from the specific dynamical organization of the accumulation–release process.

### 3.8. Statistical Organization of Energy-Release Events

**Figure A1:**
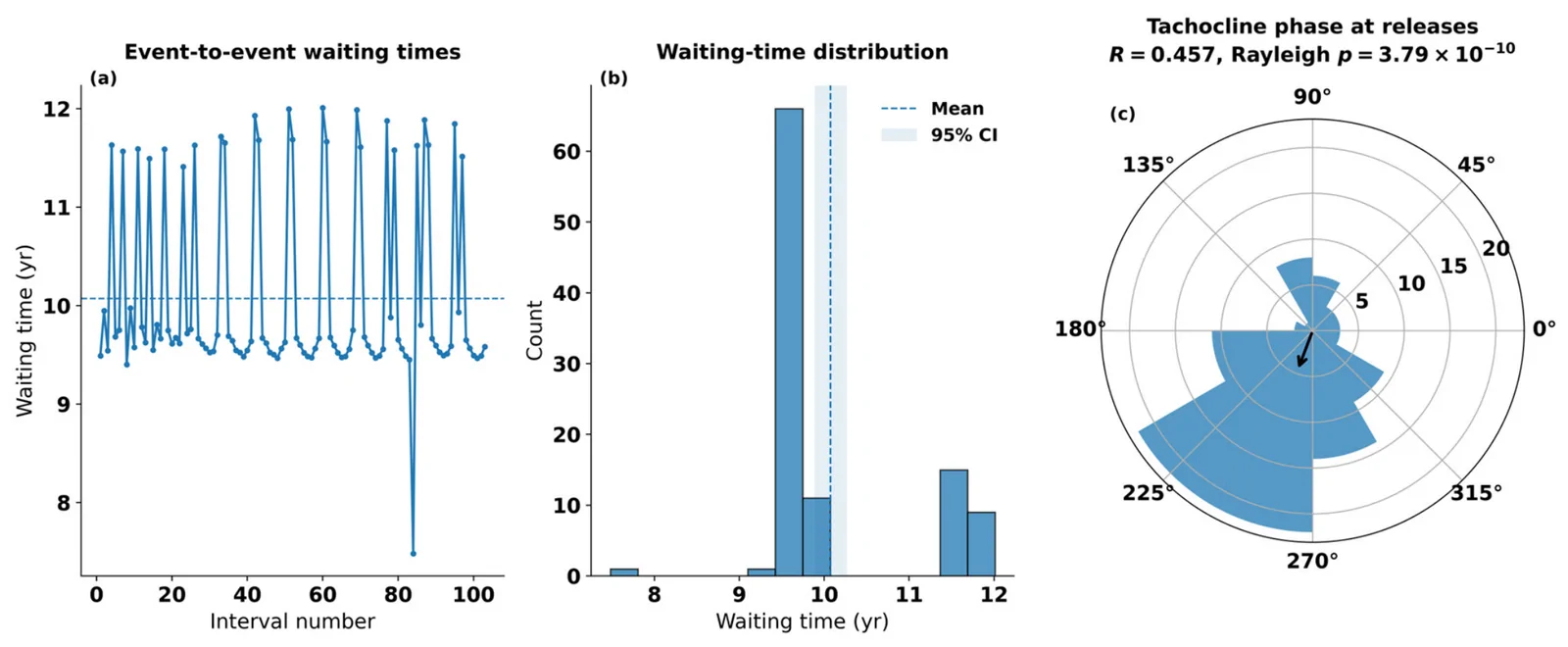
Statistical properties and tachocline-phase dependence of magnetic-energy release events. (**a**) Waiting times between successive post-transient release events. (**b**) Waiting-time distribution with the bootstrap confidence interval of the mean. (**c**) Tachocline-phase distribution at release events. The events exhibit a characteristic decadal recurrence interval and a statistically significant, but non-exclusive, phase preference (R = 0.457, $p=3.79\times 10^{-10}$).

**Figure A3:**
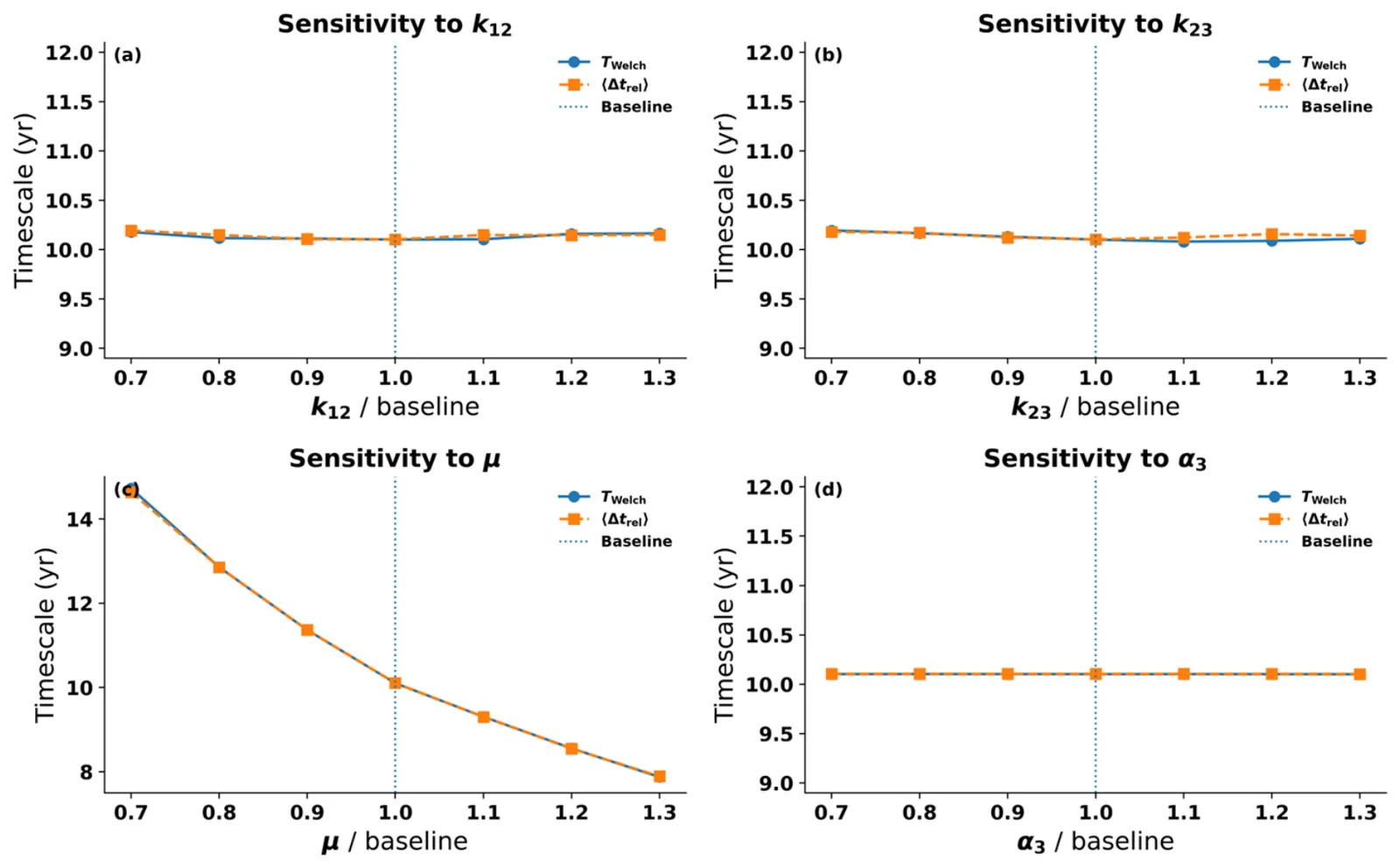
Robustness to coupling and magnetic-mode parameters. Sensitivity of the dominant Welch period and mean release interval to independent variations in (**a**) $k_{12}$, (**b**) $k_{23}$, (**c**) μ, and (**d**) $\alpha_{3}$ over 0.7–1.3 times their baseline values.

The statistical organization of the threshold-triggered releases was examined after removal of the initial 150 yr transient. The baseline trajectory contains 104 post-transient events, with a mean waiting time of approximately $\langle \Delta t_{\mathrm{rel}}\rangle ≃10.08$ yr, a median of approximately 9.64 yr, and a standard deviation of 0.93 yr. Detailed waiting-time statistics are provided in Appendix A (Figure A1).

The corresponding coefficient of variation, $\mathrm{CV}=\frac{\sigma_{\Delta t}}{\langle \Delta t_{\mathrm{rel}}\rangle }≃0.093$, indicates relatively regular recurrence with finite event-to-event variability.

Bootstrap resampling gives a 95% confidence interval of approximately 9.89–10.27 yr for the mean waiting time. This interval contains the independently estimated spectral period, $T_{\mathrm{Welch}}$ = 10.085 yr, supporting agreement between the characteristic release recurrence and the dominant collective timescale in the baseline regime.

The waiting-time distribution is therefore concentrated around the decadal scale but is not strictly periodic. Individual release intervals fluctuate around their mean because each threshold crossing depends on the continuously evolving state of all three coupled modes. The release sequence is thus better characterized as a statistically organized recurrence than as an exactly periodic event train.

These results complement the spectral analysis: the decadal organization is present both in the continuous magnetic activity and in the statistics of dynamically defined energy-release events. As demonstrated in Section 3.6, however, this correspondence is regime dependent and can break down through event skipping.

The individual waiting times fluctuate around the decadal mean without forming an exactly periodic sequence (Figure A1a). Their distribution is concentrated primarily near 9.4–10.0 yr, with a smaller group of longer intervals near 11.5–12.0 yr and an isolated shorter interval (Figure A1b). Thus, the release sequence exhibits a well-defined characteristic recurrence time while retaining finite event-to-event variability.

### 3.9. Tachocline-Phase Dependence of Energy Release

The timing of the release events was further examined relative to the instantaneous phase of the tachocline-related mode. The phase $\phi_{2}$ was evaluated immediately before each threshold crossing, as defined in Section 2.6. The resulting circular distribution is clearly nonuniform (Figure A1c).

The mean resultant length is R = 0.457, indicating a moderate concentration of release events within a preferred phase sector. The circular mean phase is ${\bar{\phi }}_{2}≃4.349\;$ rad ≃249.2°. The Rayleigh test strongly rejects phase uniformity, $p=3.79\times 10^{-10}$.

Thus, threshold crossings are statistically more likely during particular phases of the tachocline-related mode.

The concentration is nevertheless incomplete R < 0.5; release events occupy a broad phase range rather than occurring at a unique phase. The result should therefore be interpreted as a statistically significant phase preference, rather than strict phase locking. This is consistent with the hybrid mechanism: a release occurs only when the magnetic energy reaches $E_{\mathrm{crit}}$, while the tachocline mode modulates the energy transfer into the magnetic subsystem.

Together with the waiting-time statistics in Figure A1a,b, the phase distribution indicates that the release sequence is neither exactly periodic nor phase independent. Instead, it exhibits statistical temporal organization combined with a moderate preference for a particular tachocline-phase sector.

### 3.10. Local Cycle-to-Cycle and Release-Interval Variability

The close agreement between the dominant spectral period and the mean release interval does not necessarily imply a one-to-one correspondence at the level of individual cycles. To test this possibility, individual activity-cycle intervals were compared with the corresponding local release intervals. The detailed comparison is presented in Appendix D (Figure A8).

The scatter shows no systematic relation between the two quantities. The Pearson correlation coefficient is r = –0.106, p = 0.288, while the Spearman rank correlation gives ρ = 0.074, p = 0.456.

Thus, neither linear nor monotonic correlation is statistically significant. As shown in Figure A8, release intervals remain concentrated mainly around 9.5–10 yr, with a secondary group near 11.5–12 yr, whereas the corresponding local cycle intervals span a substantially broader range.

This result distinguishes global timescale organization from local cycle-to-cycle synchronization. Although $T_{\mathrm{Welch}}\approx \langle \Delta t_{\mathrm{rel}}\rangle$ for the baseline regime, individual variations in cycle duration do not predict the corresponding release interval. The agreement therefore represents a long-term statistical property of the hybrid dynamics rather than strict event-by-event synchronization.

Together with the event-skipping behavior identified in Section 3.6, this result confirms that spectral and event-based timescales provide complementary descriptions of the system and should not be treated as locally interchangeable.

### 3.11. Energetic and Numerical Validation of the Hybrid Dynamics

**Figure A4:**
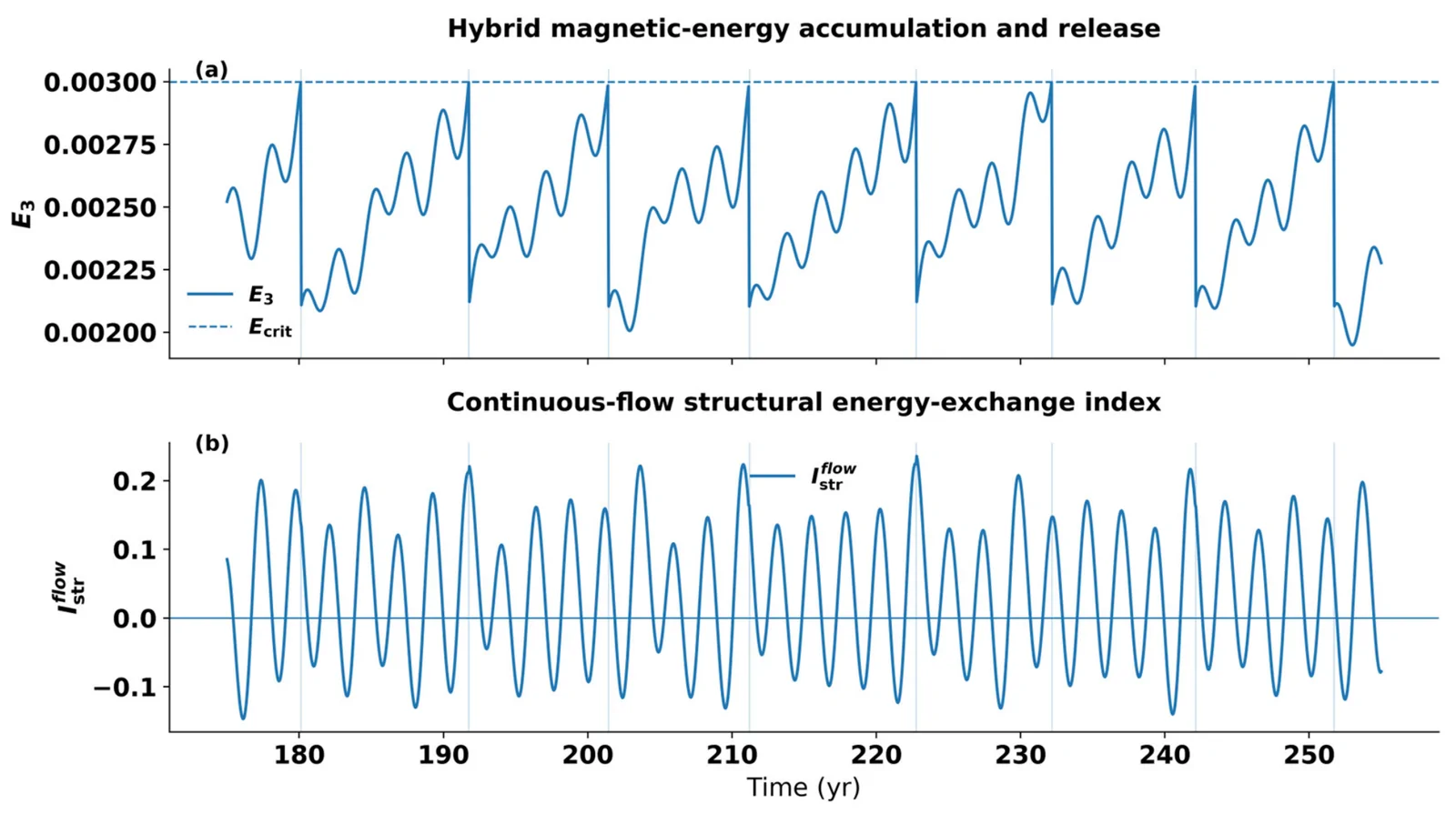
Magnetic-energy evolution and structural energy-exchange diagnostic. (**a**) Hybrid magnetic-energy accumulation and threshold-triggered release. (**b**) Continuous-flow structural energy-exchange index $I_{\mathrm{str}}^{\mathrm{flow}}$, evaluated excluding discrete reset discontinuities.

**Figure A5:**
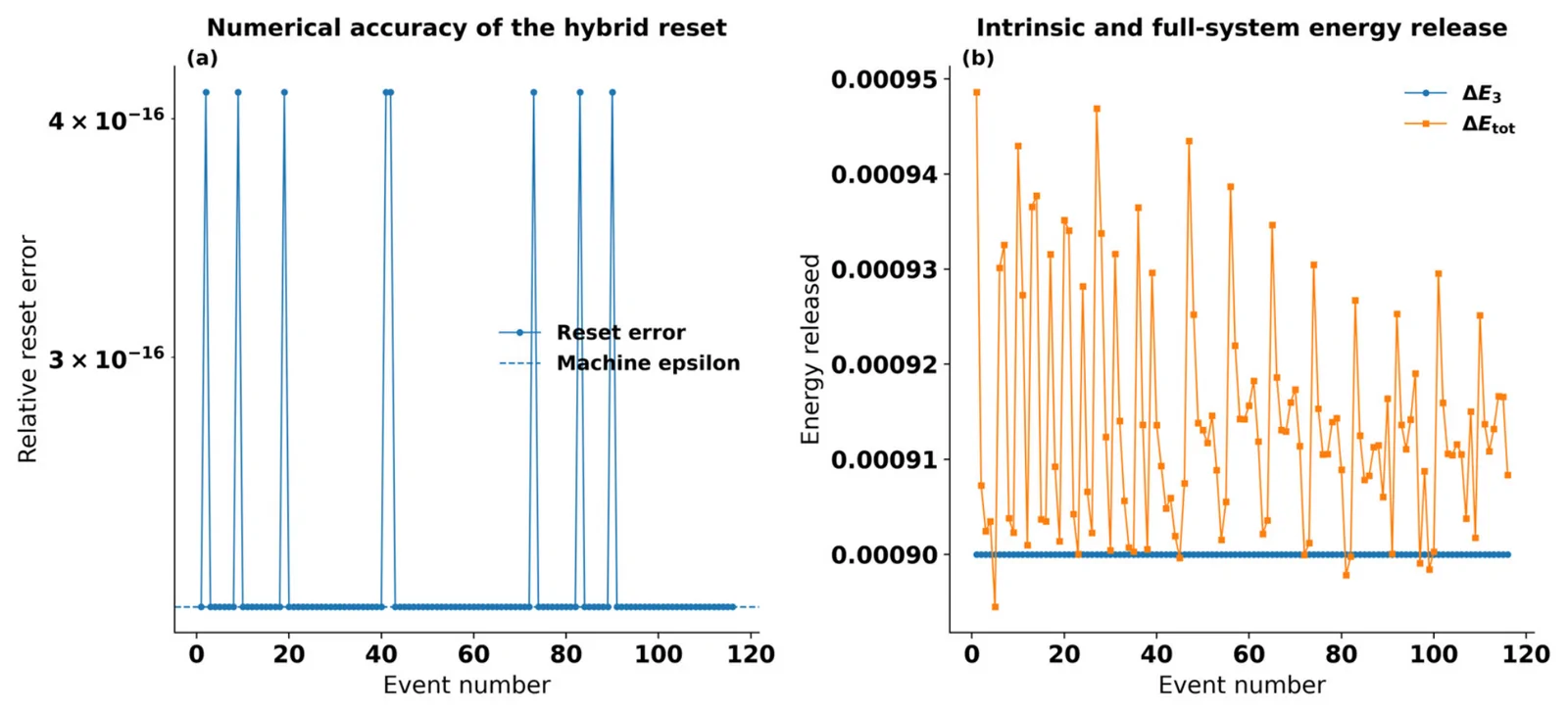
Numerical validation of the hybrid reset. (**a**) Relative error of the prescribed post-release magnetic-energy condition for all release events. (**b**) Intrinsic magnetic-energy reduction $\Delta E_{3}$ and corresponding full-system energy change $\Delta E_{\mathrm{tot}}$. The reset condition is satisfied to near machine precision, while the small variability of $\Delta E_{\mathrm{tot}}$ reflects changes in the interaction energies.

**Figure A6:**
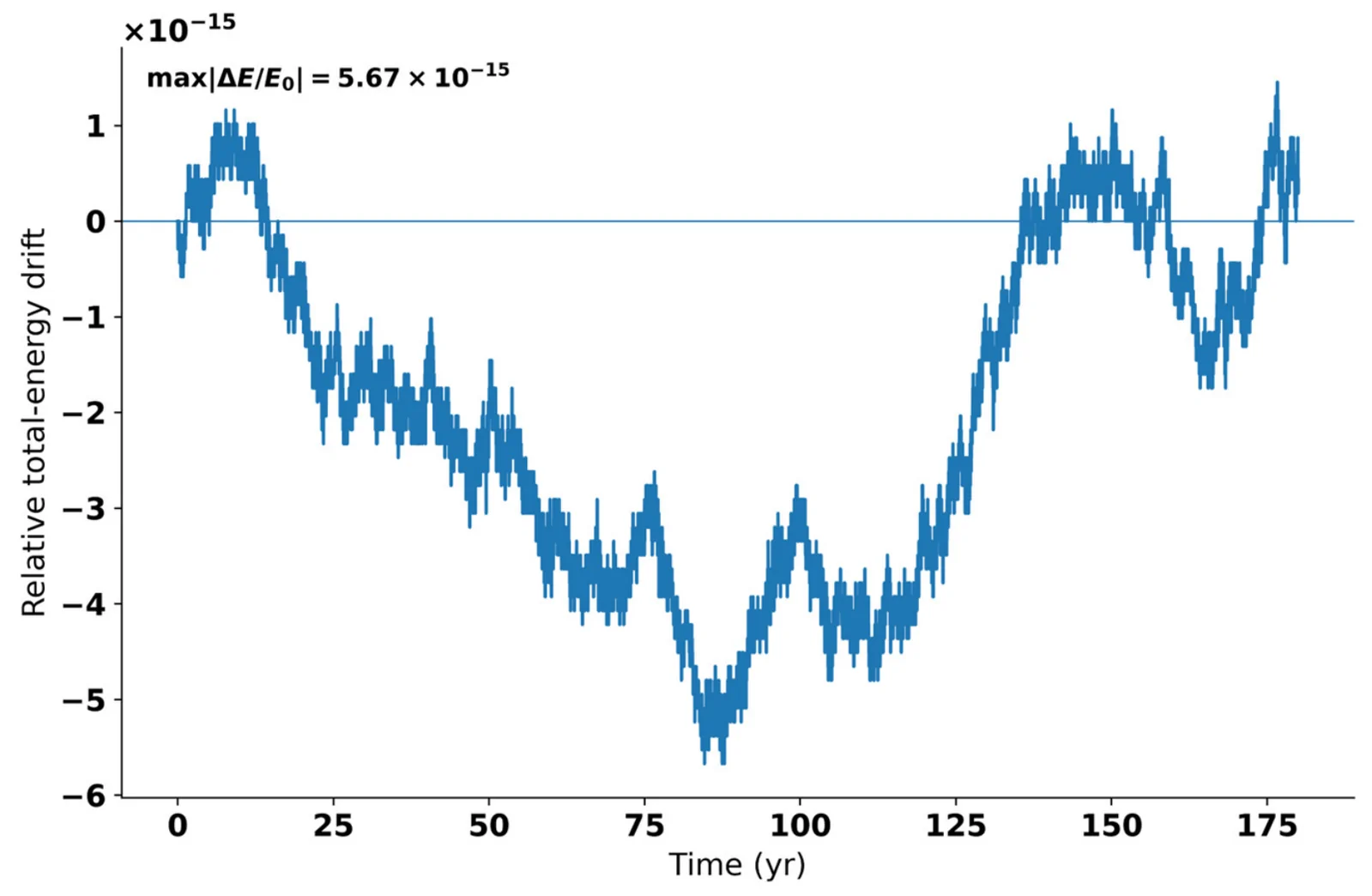
Conservative-limit numerical validation. Relative drift of the total energy during conservative evolution. The maximum relative deviation, $5.67\times 10^{-15}$, demonstrates conservation of the analytically defined total energy to near machine precision.

**Figure A7:**
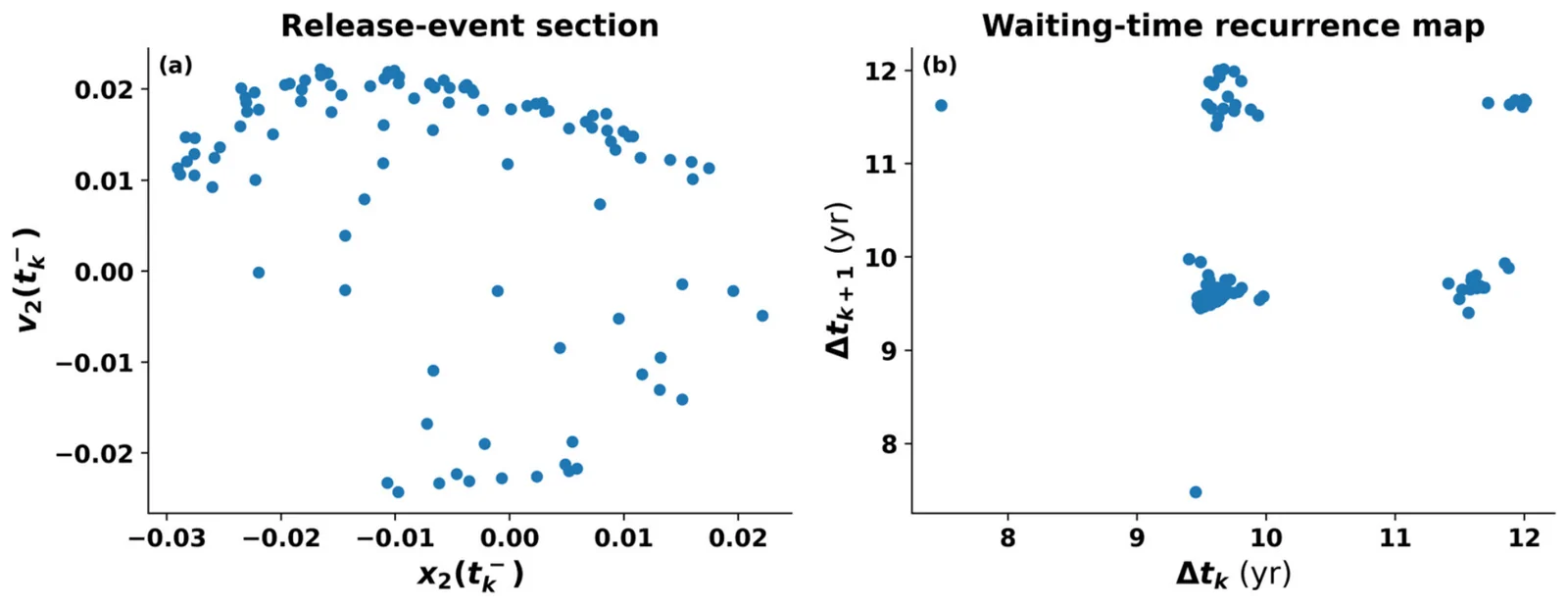
Event-based dynamical diagnostics. (**a**) Pre-release states of the tachocline subsystem in the ($x_{2}$, $v_{2}$) plane at successive magnetic-energy threshold crossings. (**b**) Waiting-time recurrence map $\Delta t_{k}$ versus $\Delta t_{k+1}$. The event states and recurrence intervals exhibit structured but finite variability within the persistent hybrid regime.

**Figure A8:**
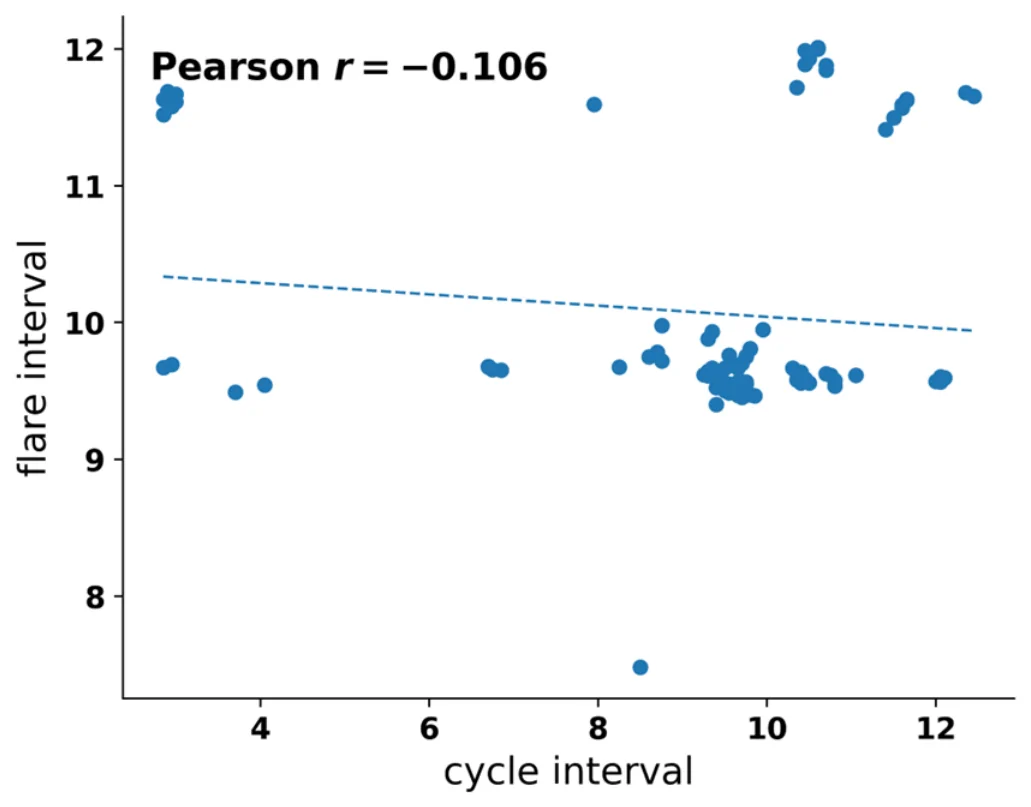
Local cycle-to-cycle and release-interval variability. Comparison of individual activity-cycle intervals with neighboring magnetic-energy release intervals.

**Figure A12:**
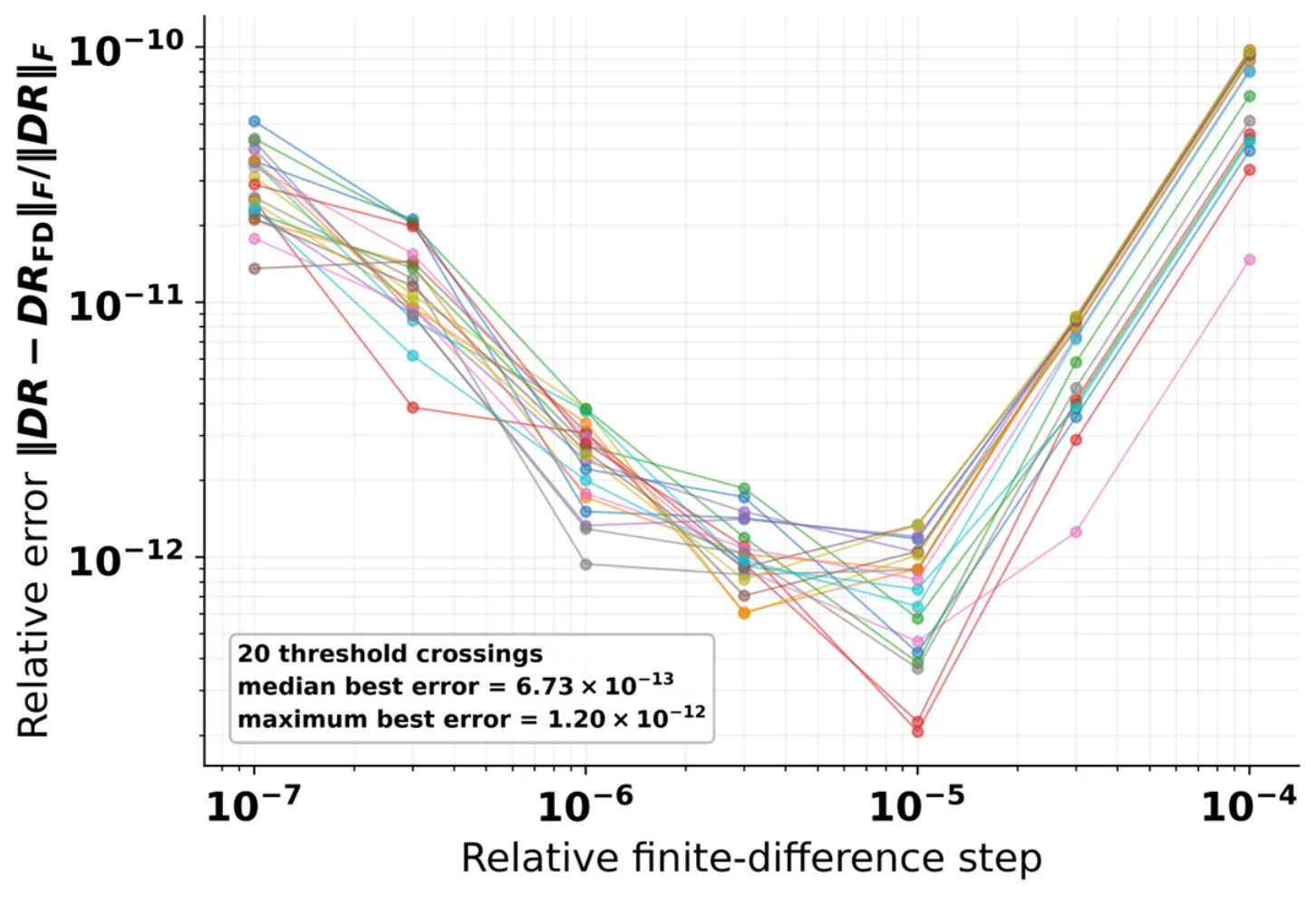
Finite-difference validation of the state-dependent reset Jacobian. Relative Frobenius discrepancy between the analytical reset Jacobian and an independent central finite-difference approximation of the complete nonlinear reset map, evaluated at 20 actual upward threshold crossings of the baseline trajectory. Each curve represents one threshold crossing, and the relative finite-difference step is varied over several orders of magnitude. The characteristic minimum at intermediate step sizes reflects the balance between finite-difference truncation and floating-point errors. The median and maximum best relative errors across the tested crossings were $6.73\times 10^{-13}$ and $1.20\times 10^{-12}$, respectively.

The energetic consistency and numerical accuracy of the hybrid formulation were verified independently from the spectral and event-statistical analyses. The validation addressed three aspects of the model: the continuous-flow energy exchange, the accuracy of the discrete reset operator, and conservation of the total energy in the conservative limit. Additional event-based phase-space diagnostics were used to characterize the organization of successive threshold crossings. Detailed results are presented in Appendix C and Appendix D (Figure A4, Figure A5, Figure A6 and Figure A7).

The continuous-flow magnetic energy exhibits repeated accumulation toward $E_{\mathrm{crit}}$, followed by discrete release and subsequent recovery (Figure A4a). The structural energy-exchange index $I_{\mathrm{str}}^{\mathrm{flow}}$ alternates between positive and negative values during these intervals (Figure A4b), reflecting successive phases of magnetic-energy gain and loss. Because the index is evaluated only along continuous-flow segments, the discontinuous reset itself is excluded from this diagnostic.

The accuracy of the discrete reset was tested directly at every release event (Figure A5a). The prescribed post-release target (Equation (5)) is reproduced with a maximum relative error of $\varepsilon_{\max}=4.13\times 10^{-16}$, i.e., at approximately machine precision. For the baseline parameters, the intrinsic magnetic-energy release is fixed by the reset rule at $\Delta E_{3}=\eta E_{\mathrm{crit}}=9.0\times 10^{-4}$.

The change in the full-system energy, $\Delta E_{\mathrm{tot}}$, differs slightly from $\Delta E_{3}$ (Figure A5b) because resetting $x_{3}$ also changes the interaction energies $V_{23}$ and $V_{\beta }$. This distinction confirms that the intrinsic magnetic-energy decrement and the total energetic effect of an event should not be identified.

A separate conservative-limit calculation was performed with continuous dissipation removed and the hybrid release mechanism disabled. The total energy remained conserved to numerical precision, with $\underset{t}{\max}\left|\frac{E_{\mathrm{tot}}(t)-E_{\mathrm{tot}}(0)}{E_{\mathrm{tot}}(0)}\right|=5.67\times 10^{-15}$ (Figure A6). This provides an independent check of both the reciprocal coupling formulation and the numerical integration.

Finally, the event-defined phase-space section and waiting-time recurrence map provide complementary diagnostics of the established hybrid regime (Figure A7). The release states occupy a structured region of the ($x_{2}$,$v_{2}$) plane rather than a single point (Figure A7a), while successive waiting times form several compact groups (Figure A7b). These patterns are consistent with organized but non-identical release cycles and complement the statistical and phase analyses presented above. They are used here as descriptive event-based diagnostics rather than, by themselves, as evidence for periodicity, quasiperiodicity, or chaos.

Overall, these tests show that the reported hybrid dynamics are consistent with the prescribed energy balance, accurately resolve the discrete reset, and recover energy conservation in the conservative limit. The dynamical stability of the resulting hybrid regime is examined separately through the saltation-based Lyapunov analysis.

### 3.12. Lyapunov Characterization of the Hybrid Regime

**Figure 7:**
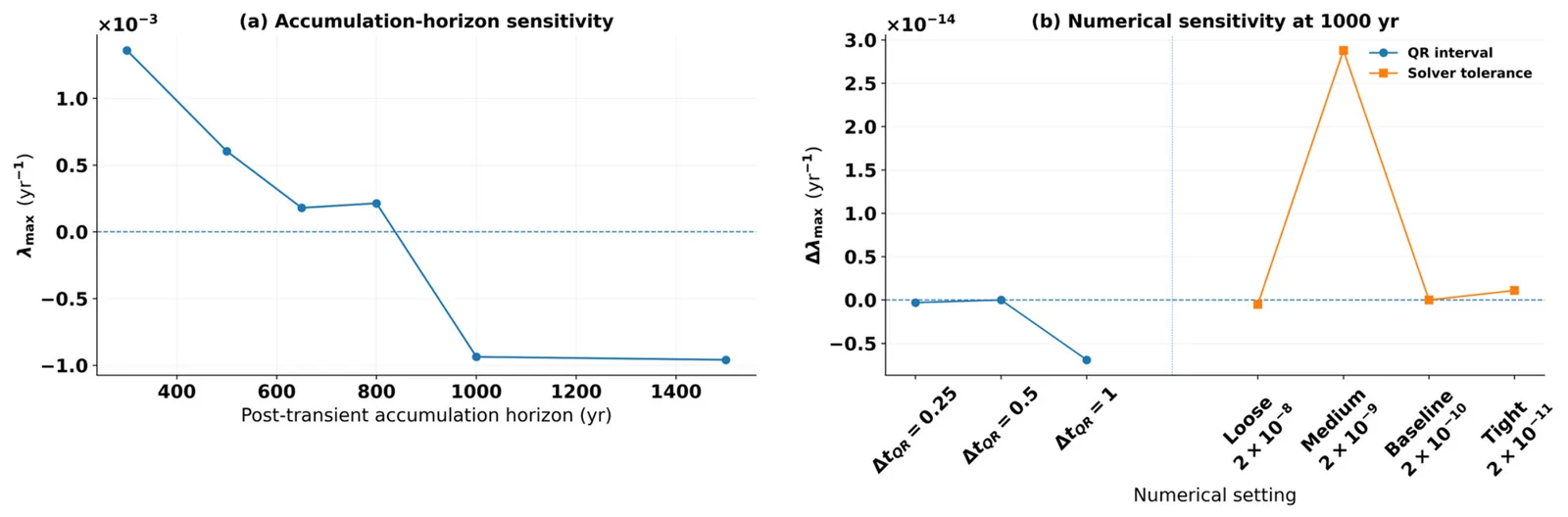
Finite-time convergence and numerical sensitivity of the largest hybrid Lyapunov exponent for the baseline regime. (**a**) Dependence of the largest finite-time Lyapunov exponent $\lambda_{\max}$ on the post-transient accumulation horizon. Small positive estimates obtained at intermediate horizons do not persist as the accumulation time is increased; $\lambda_{\max}$ becomes negative at 1000 yr and remains negative at 1500 yr. (**b**) Numerical sensitivity of the 1000 yr estimate to the QR-renormalization interval and solver tolerances. The dashed horizontal lines denote zero.

The dynamical character of the hybrid system was examined using the saltation-corrected Lyapunov formulation described in Section 2.8. For the baseline parameter set, the largest finite-time Lyapunov exponent exhibited a pronounced dependence on the accumulation horizon (Figure 7a). The estimates decreased from $1.36\times 10^{-3}\;{\mathrm{yr}}^{-1}$ at 300 yr to $6.04\times 10^{-4}\;{\mathrm{yr}}^{-1}$ at 500 yr and $1.80\times 10^{-4}\;{\mathrm{yr}}^{-1}$ at 650 yr. At longer horizons, the estimate became negative, reaching $-9.36\times 10^{-4}\;{\mathrm{yr}}^{-1}$ at 1000 yr and $-9.59\times 10^{-4}\;{\mathrm{yr}}^{-1}$ at 1500 yr. Thus, the small positive values obtained at intermediate integration times do not persist for the baseline regime.

To determine whether this horizon dependence could be attributed to numerical settings, the QR-renormalization interval and solver tolerances, expressed as $\Delta \lambda_{\max}=\lambda_{\max}-\lambda_{\max^{\mathrm{ref}}}$, where $\lambda_{\max^{\mathrm{ref}}}$ is the baseline 1000 yr estimate obtained with $\Delta t_{\mathrm{QR}}$ = 0.5 yr, $rtol=2\times 10^{-10}$, and $atol=2\times 10^{-12}$, were varied independently (Figure 7b). The resulting variations in $\lambda_{\max}$ were negligible, indicating that the observed horizon dependence reflects finite-time convergence rather than sensitivity to the QR cadence or integration tolerances. The negligible numerical variations in panel (b), compared with the horizon dependence in panel (a), indicate that the observed evolution of $\lambda_{\max}$ is a finite-time convergence effect rather than an artifact of QR cadence or integration accuracy.

At the 1000 yr post-transient accumulation horizon, the baseline spectrum was λ= (−0.000936, −0.001195, −0.002738, −0.005929, −0.007146, −0.007786) yr^−1^. Changing the QR interval from 0.25 to 1.0 yr produced a total spread of only $6.95\times 10^{-15}{\mathrm{yr}}^{-1}$, while varying the integration tolerances over three orders of magnitude produced a spread of $2.93\times 10^{-14}{\mathrm{yr}}^{-1}$. The evolution of $\lambda_{\max}$ with accumulation horizon therefore cannot be attributed to the QR cadence or solver accuracy.

**Figure 8:**
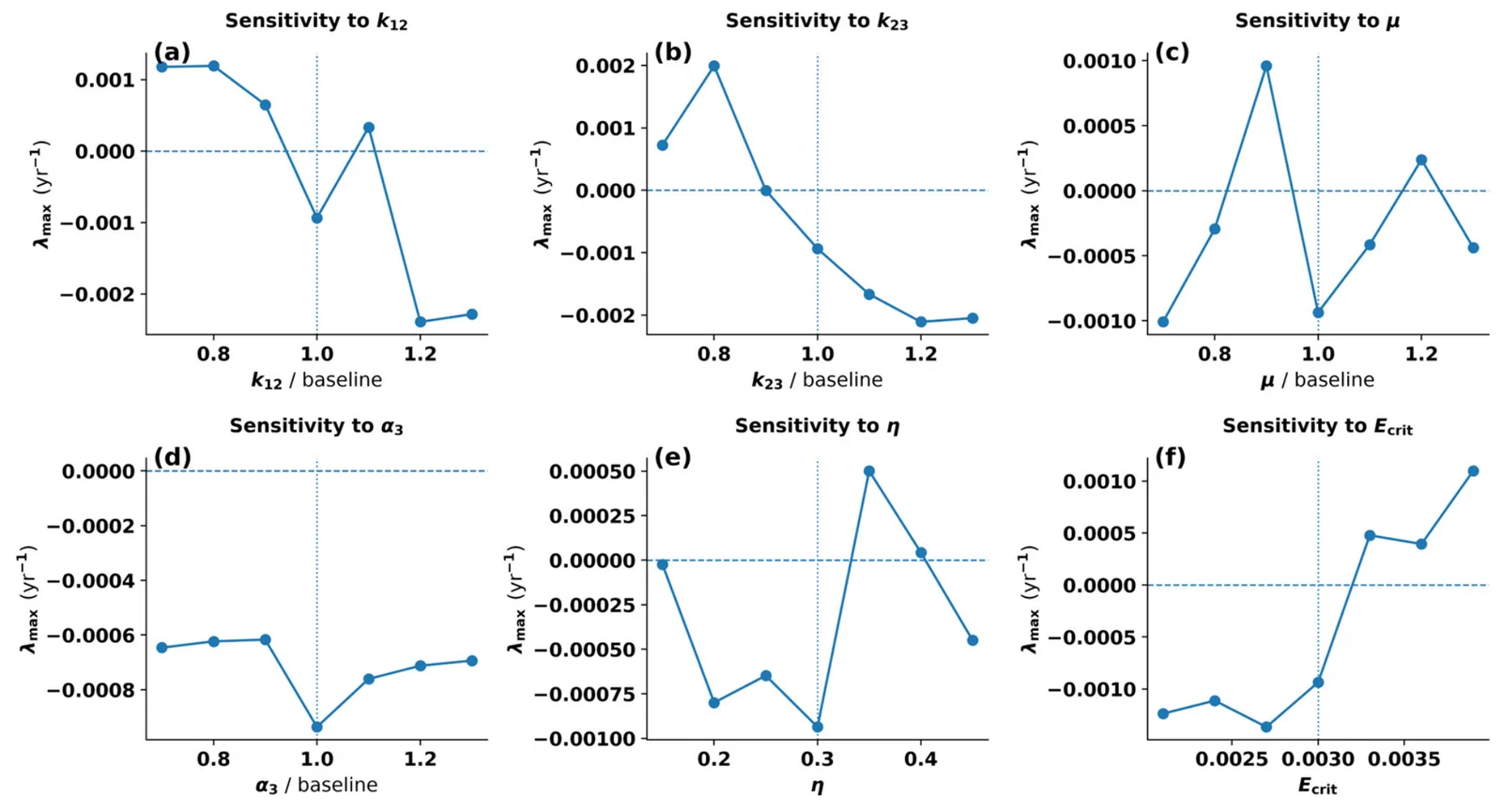
Parameter sensitivity of the finite-time largest hybrid Lyapunov exponent. Largest finite-time Lyapunov exponent $\lambda_{\max}$ obtained for one-factor variations in (**a**) $k_{12}$, (**b**) $k_{23}$, (**c**) μ, (**d**) $\alpha_{3}$, (**e**) the release depth η, and (**f**) the critical magnetic-energy threshold $E_{\mathrm{crit}}$. Vertical dotted lines indicate the baseline parameter values, and horizontal dashed lines denote $\lambda_{\max}=0$.

To examine whether this behavior is specific to the baseline configuration, the Lyapunov analysis was extended to one-factor variations in the principal coupling, nonlinear, and release parameters. All parameter cases shown in Figure 8 were evaluated using a common 1000 yr post-transient accumulation horizon. Both positive and negative finite-time largest-exponent estimates occur within the investigated parameter neighborhood. Sign changes are observed under variations in $k_{12}$, $k_{23}$, μ, η, and $E_{\mathrm{crit}}$, whereas all tested variations of $\alpha_{3}$ yield negative finite-time estimates.

The strongest positive finite-time values occur for reduced $k_{12}$ and $k_{23}$, with the largest value reaching approximately $2.0\times 10^{-3}\;{\mathrm{yr}}^{-1}$ for $k_{23}/k_{23,0}=0.8$. The response to $E_{\mathrm{crit}}$ also shows a transition from negative to positive finite-time estimates as the threshold is increased above its baseline value. In contrast, variations in $\alpha_{3}$ over the investigated range produce comparatively weak changes and do not result in a sign reversal.

**Figure A13:**
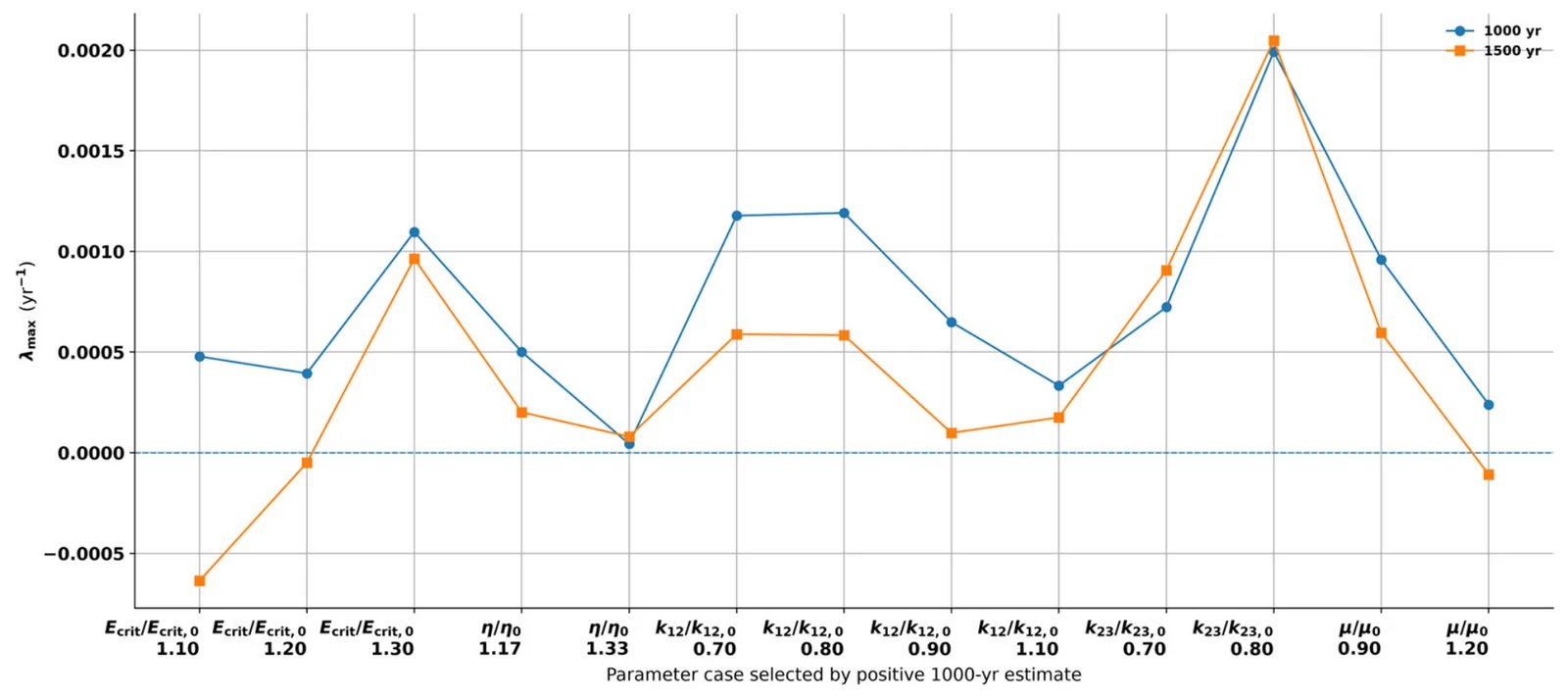
Extended-horizon validation of positive finite-time Lyapunov estimates. Comparison of the largest finite-time hybrid Lyapunov exponent $\lambda_{\max}$ obtained using 1000 and 1500 yr post-transient accumulation horizons. Only parameter configurations with $\lambda_{\max}>0$ in the original 1000 yr sweep were selected for the extended calculation. Ten of the 13 selected configurations retained positive estimates at 1500 yr, whereas three changed sign. The horizontal dashed line denotes $\lambda_{\max}=0$. Persistent positive finite-time estimates are interpreted as evidence of finite-time exponential sensitivity and not, by themselves, as definitive evidence of asymptotic chaos.

Because the baseline analysis demonstrates that the sign of a finite-time Lyapunov estimate can depend on the accumulation horizon, the positive values in Figure 8 were subjected to an additional extended-horizon test. All 13 parameter configurations with $\lambda_{\max}>0$ at 1000 yr were recomputed using a 1500 yr accumulation horizon. Ten remained positive, whereas three changed sign; the complete extended-horizon comparison is provided in Appendix F (Figure A13).

Persistent positive finite-time estimates occurred for four variations of $k_{12}$, two variations of $k_{23}$, two values of η, one value of μ, and one value of $E_{\mathrm{crit}}$. These results demonstrate that the hybrid system exhibits parameter-dependent finite-time exponential sensitivity, but the positive estimates are not interpreted as definitive evidence of asymptotic chaos.

## 4. Discussion

### 4.1. Emergent Origin of the Decadal Collective Timescale

Reduced-order nonlinear models can reveal collective timescales that do not coincide with the intrinsic periods of their individual components [29,30,31]. In solar-dynamo studies, such models have likewise demonstrated that nonlinear interactions among a small number of effective degrees of freedom can produce cyclic modulation and long-term variability [21], while remaining complementary to spatially resolved dynamo theory [18].

An alternative approach to cycle selection is provided by externally synchronized dynamo models. For example, Ref. [32] considered a Tayler–Spruit-type solar dynamo in which the α-effect is periodically modulated by an assumed planetary tidal forcing with an approximately 11.07 yr periodicity, allowing the magnetic cycle to synchronize with the external modulation. In contrast, the present model contains no external periodic forcing. The approximately decadal timescale therefore arises autonomously from the nonlinear coupling and hybrid accumulation–release dynamics rather than from synchronization to a prescribed external clock.

The present results show that the approximately decadal timescale is not simply inherited from the prescribed tachocline-related period $T_{2}$ = 11 yr. The baseline values, $T_{\mathrm{FFT}}$ = 10.084 yr and $T_{\mathrm{Welch}}$ = 10.085 yr, remain nearly unchanged under substantial variations in the intrinsic modal periods. In particular, $T_{2}$ = 8 and 14 yr yield $T_{\mathrm{Welch}}\approx 10.31$ and 10.29 yr, respectively. Moreover, under simultaneous ±20% perturbations of $T_{1}$, $T_{2}$, $T_{3}$, all 24 realizations retain dominant periods within 9–12 yr, with a mean of 10.21±0.26 yr.

In contrast, the collective timescale is substantially more sensitive to parameters governing magnetic accumulation and recovery. Increasing the release depth from η = 0.20 to 0.40 shifts $T_{\mathrm{Welch}}$ from approximately 6.44 to 14.72 yr, whereas varying μ from 0.7 $\mu_{0}$ to 1.3 $\mu_{0}$ changes it from approximately 14.69 to 7.88 yr. Variations in $k_{12},$ $k_{23},$ and $\alpha_{3}$ produce much smaller changes.

Thus, the decadal timescale is best interpreted as an emergent collective property of the coupled hybrid dynamics, rather than as an imposed or directly inherited 11 yr clock. This does not imply complete independence from the intrinsic periods; more generally, $T_{\mathrm{collective}}=F(T_{1},T_{2},T_{3},\eta ,\mu ,E_{\mathrm{crit}},k_{ij},\alpha_{i},\beta ,\ldots )$.

Within the investigated parameter neighborhood, however, η and μ exert substantially stronger control than the prescribed modal periods. The localized event-skipping regime further indicates that $T_{2}$ can modulate threshold-crossing dynamics without acting as a direct pacemaker of the collective cycle. This points to the accumulation–release process as the principal timescale-selection mechanism examined in the following section.

### 4.2. Accumulation–Release Dynamics as the Timescale-Selection Mechanism

The parameter sensitivity indicates that the collective timescale is governed primarily by the magnetic accumulation–release process rather than by an individual intrinsic modal period. Nonlinear coupling, amplitude-dependent dissipation, and nonlinear stiffness can modify characteristic oscillator timescales [29,30,31]; in the present hybrid system, threshold-triggered energy release introduces an additional recovery timescale.

From an energetic viewpoint, the continuous dynamics can be interpreted in terms of redistribution of energy among interacting generalized degrees of freedom, consistent with the standard mechanical description of coupled systems [45]. In the present hybrid model, when the magnetic energy reaches $E_{\mathrm{crit}}$, the reset reduces it according to Equation (5), after which the coupled dynamics replenish the magnetic subsystem until the next threshold crossing. The release depth η therefore determines the post-release energy deficit, whereas the Van der Pol parameter μ controls the rate of magnetic self-excitation and recovery. Their strong influence on the dominant period supports the schematic relation $T_{\mathrm{collective}}\sim T_{\mathrm{recharge}}=F(\eta ,\mu ;T_{i},k_{ij},E_{\mathrm{crit}},\alpha_{i},\beta ,\ldots )$.

The comparison of the three release formulations further supports this interpretation. Without release, magnetic energy evolves toward a high-energy state, whereas continuous dissipation suppresses threshold crossings and produces threshold avoidance. Only the discrete hybrid mechanism generates repeated bounded sequences of accumulation, release, and recovery. Thus, the decadal timescale arises from the interaction between continuous nonlinear energy transfer and discrete threshold resets, rather than from either mechanism alone.

This accumulation–release interpretation is consistent with the broader role of nonlinear feedback and energy redistribution in reduced nonlinear and dynamo models [18,21,30,46,47,48]. Within the present model, however, it has a specific meaning: the characteristic collective timescale is selected primarily by the time required for the magnetic subsystem to recover from a release and return to the threshold.

In this sense, the collective cycle can be viewed as an energy-recovery timescale of a structured system of interacting generalized modes: continuous dynamics redistribute energy among the modes [30,45], whereas the discrete reset periodically removes a prescribed fraction from the magnetic subsystem.

### 4.3. Spectral Organization, Event Recurrence, and Event Skipping

The hybrid regime exhibits close agreement between the dominant spectral timescale and the mean recurrence time of energy-release events. For the baseline solution, $T_{\mathrm{Welch}}\approx 10.09$ yr, while the mean post-transient waiting time is approximately 10.08 yr. The relatively small waiting-time variability (CV≈0.093) indicates statistically organized recurrence, but not an exactly periodic sequence. Such non-identical cycles are consistent with the broader behavior of nonlinear dynamical and reduced dynamo systems, where nonlinear interactions can generate modulation and temporal variability [31,46,47,48].

Importantly, this global agreement does not imply strict cycle-to-cycle synchronization. Local cycle and release intervals are not significantly correlated (Pearson r = −0.106, p = 0.288; Spearman ρ = 0.074, p = 0.456). Thus, the spectral cycle characterizes the long-term organization of the continuous dynamics, whereas threshold crossings describe a related but distinct discrete event process.

This distinction becomes particularly clear in the event-skipping regime observed near $T_{2}\approx 11.7-12.7$ yr. There, the spectral timescale remains approximately 9.4–9.8 yr, while mean release intervals can increase to 13–30 yr because some oscillatory excursions do not reach $E_{\mathrm{crit}}$. Consequently, one release event may occur only after several underlying activity cycles. The correspondence $T_{\mathrm{spectral}}\approx \langle \Delta t_{\mathrm{release}}\rangle$ is therefore a property of the baseline regime rather than a universal identity. Overall, the results indicate global spectral organization of the release process without rigid local synchronization, with event skipping providing a natural mechanism for longer and more variable release intervals.

### 4.4. Role of the Tachocline Mode in Energy-Release Timing

The tachocline-related mode plays a distinct role in the organization of energy-release events. Although variations in its intrinsic period $T_{2}$ do not directly determine the decadal collective timescale, the release events exhibit a statistically significant preference for a particular tachocline phase. For the 104 post-transient events, the mean pre-release phase is ${\bar{\phi }}_{2}$ = 4.349 rad (249.2°), with a mean resultant length R = 0.457 and a Rayleigh-test probability $p=3.79\times 10^{-10}$.

This phase preference is consistent with the tachocline mode acting as an effective mediator of energy transfer to the magnetic subsystem, rather than as a clock that directly imposes the cycle period. Such an interpretation is compatible with solar-dynamo theory, in which differential rotation and nonlinear interactions contribute to the generation and modulation of large-scale magnetic fields [18,48].

The moderate value of R, however, indicates a broad phase distribution rather than strict phase locking. The results therefore support phase-dependent modulation of threshold crossing without rigid synchronization. In this sense, the tachocline-related mode influences when magnetic-energy release is favored, while the collective recurrence timescale is determined by the coupled accumulation–release dynamics of the full system.

### 4.5. Dynamical Character of the Persistent Hybrid Regime

The dynamical characterization of the hybrid regime requires caution because spectral regularity and structured phase-space trajectories alone do not distinguish periodic, quasiperiodic, and weakly chaotic dynamics [31,46]. The saltation-corrected Lyapunov analysis shows that, for the baseline configuration, the small positive finite-time exponent obtained at intermediate horizons does not persist: $\lambda_{\max}$ becomes negative at 1000 yr and remains negative at 1500 yr. Its negligible sensitivity to the QR interval and solver tolerances indicates that this behavior reflects finite-time convergence rather than numerical settings.

The broader parameter analysis reveals a more complex picture. Variations in $k_{12}$, $k_{23}$, μ, η, and $E_{\mathrm{crit}}$ produce both positive and negative finite-time estimates. Of the 13 configurations with $\lambda_{\max}>0$ at 1000 yr, 10 remain positive at 1500 yr, indicating persistent finite-time exponential sensitivity in parts of the investigated parameter neighborhood.

Accordingly, a single dynamical classification is not assigned to the entire parameter domain. The baseline regime shows no robust positive largest Lyapunov exponent over the investigated horizons, whereas several neighboring configurations retain positive finite-time estimates. These cases are not interpreted as definitive evidence of asymptotic chaos, which would require longer integrations and dedicated hybrid bifurcation analysis. Likewise, the baseline result alone does not establish quasiperiodicity [31,46].

### 4.6. Physical Interpretation, Scope, and Limitations

The present three-mode model should be interpreted as a reduced phenomenological representation of collective solar dynamics rather than as a spatially resolved solar-dynamo model. The generalized coordinates $x_{1}$, $x_{2}$, and $x_{3}$ represent, respectively, a slow internal mode, a tachocline-associated energy-transfer mode, and a collective magnetic subsystem. This type of low-order description is useful for isolating nonlinear mechanisms and characteristic timescales, but necessarily omits much of the spatial and MHD complexity of the solar dynamo [13,18,47,48].

Accordingly, the model parameters and modal energies are effective quantities expressed in normalized units and should not be identified directly with measured solar properties. In particular, the threshold-reset mechanism represents a generic threshold-triggered magnetic-energy release rather than a detailed model of an individual solar flare. Quantitative conversion of $E_{3}$, $E_{\mathrm{crit}}$, or the released energy into physical units would require independent calibration against observations or MHD simulations.

The Van der Pol–Duffing representation is likewise not unique. It was selected because it provides a compact framework combining self-sustained dynamics, amplitude regulation, nonlinear frequency shifts, and explicit inter-mode energy exchange [29,30,31]. Alternative reduced descriptions could employ other nonlinear oscillators or coupling laws. Therefore, the main physical conclusion concerns the collective mechanism demonstrated here: nonlinear inter-mode energy transfer combined with threshold-mediated accumulation and release can autonomously generate a robust decadal timescale without external periodic forcing.

The structural energy-exchange index used here retains the energy-rate interpretation underlying the D-index framework [39], but is deliberately referred to as an index rather than an entropy because it measures the normalized rate of magnetic-energy change and is not a thermodynamic state function.

Several limitations remain. The present analysis does not derive the reduced parameters from first-principles solar MHD, does not include spatial structure or stochastic fluctuations, and does not establish a direct quantitative correspondence between individual model release events and observed solar eruptions. Moreover, the Lyapunov analysis does not provide evidence for a robust positive largest Lyapunov exponent in the baseline regime over the investigated accumulation horizons. This result should not be interpreted as establishing a specific asymptotic dynamical class, since distinguishing among periodic, quasiperiodic, and more weakly unstable hybrid dynamics would require additional long-time and bifurcation analyses [31,46]. Future work should therefore focus on calibration against observed magnetic and activity indices, estimation of effective parameters from observations or MHD simulations, and testing more physically detailed or stochastic energy-release mechanisms.

The present reset rule is also not intended to reproduce the broad observed distribution of solar-flare energies. Because both $E_{\mathrm{crit}}$ and η are fixed, the intrinsic magnetic-mode energy decrement at each release is approximately constant, $\Delta E_{3}^{\mathrm{rel}}=\eta E_{\mathrm{crit}}$. The current model therefore addresses the timing and recurrence of release events rather than their observed energy-size distribution. A natural extension would allow $E_{\mathrm{crit}}$ and/or η to depend on the system state or include stochastic variability, thereby producing a distribution of release magnitudes.

## 5. Conclusions

We developed an autonomous nonlinear three-mode hybrid model in which a slow internal mode, a tachocline-related mode, and a magnetic mode interact through reciprocal linear and nonlinear coupling. Magnetic-energy accumulation is terminated by a threshold-triggered discrete release, providing a minimal phenomenological representation of accumulation–transfer–release dynamics without external periodic forcing.

The model generates a persistent approximately decadal collective timescale that is not a direct copy of any single prescribed intrinsic modal period. This timescale remains comparatively stable under variations in the intrinsic periods, initial conditions, coupling parameters, and post-processing settings, while the release depth and magnetic-mode regulation exert stronger control over the recurrence timescale. The results therefore support the interpretation of the decadal variability as an emergent property of the coupled hybrid dynamics rather than a directly imposed oscillation period.

The saltation-corrected Lyapunov analysis further reveals a parameter-dependent dynamical structure. For the baseline configuration, the small positive finite-time largest exponent obtained at intermediate horizons does not persist at longer accumulation times, and no robust positive $\lambda_{\max}$ is detected over the investigated horizons. In contrast, several neighboring parameter configurations retain positive finite-time estimates up to 1500 yr, indicating persistent finite-time exponential sensitivity. These results do not by themselves establish either asymptotic chaos in the positive cases or quasiperiodicity of the baseline regime.

Overall, the proposed framework demonstrates how a robust collective timescale and organized energy-release recurrence can emerge from nonlinear inter-mode energy exchange combined with threshold-mediated release. The model should therefore be regarded as a reduced phenomenological framework for investigating timescale selection and regime organization in nonlinear solar dynamics, rather than as a unique or quantitatively calibrated representation of the solar interior. Future work should connect the effective variables and release statistics more directly to observations and examine the parameter-dependent dynamical transitions using dedicated hybrid bifurcation analysis.

## Figures and Tables

**Table 1:** Baseline parameters of the three-mode hybrid model. Time is expressed in years; the remaining quantities are given in the normalized units of the reduced model.

Parameter	Baseline Value	Role
$T_{1}$	22 yr	intrinsic period of the slow internal mode
$T_{2}$	11 yr	intrinsic period of the tachocline-related mode
$T_{3}$	5 yr	intrinsic period of the magnetic mode
$\gamma_{1}$	0.010	damping of the slow mode
$\gamma_{2}$	0.015	damping of the tachocline-related mode
$\alpha_{1}$	0.05	Duffing nonlinearity of the slow mode
$\alpha_{2}$	0.15	Duffing nonlinearity of the tachocline-related mode
$\alpha_{3}$	0.40	Duffing nonlinearity of the magnetic mode
$k_{12}$	0.035	linear coupling between modes 1 and 2
$k_{23}$	0.140	linear coupling between modes 2 and 3
β	0.55	nonlinear tachocline–magnetic coupling
μ	0.035	Van der Pol self-excitation/dissipation coefficient
$E_{\mathrm{crit}}$	0.0030	magnetic-energy release threshold
η	0.30	fractional magnetic-energy release depth

**Figure A2:**
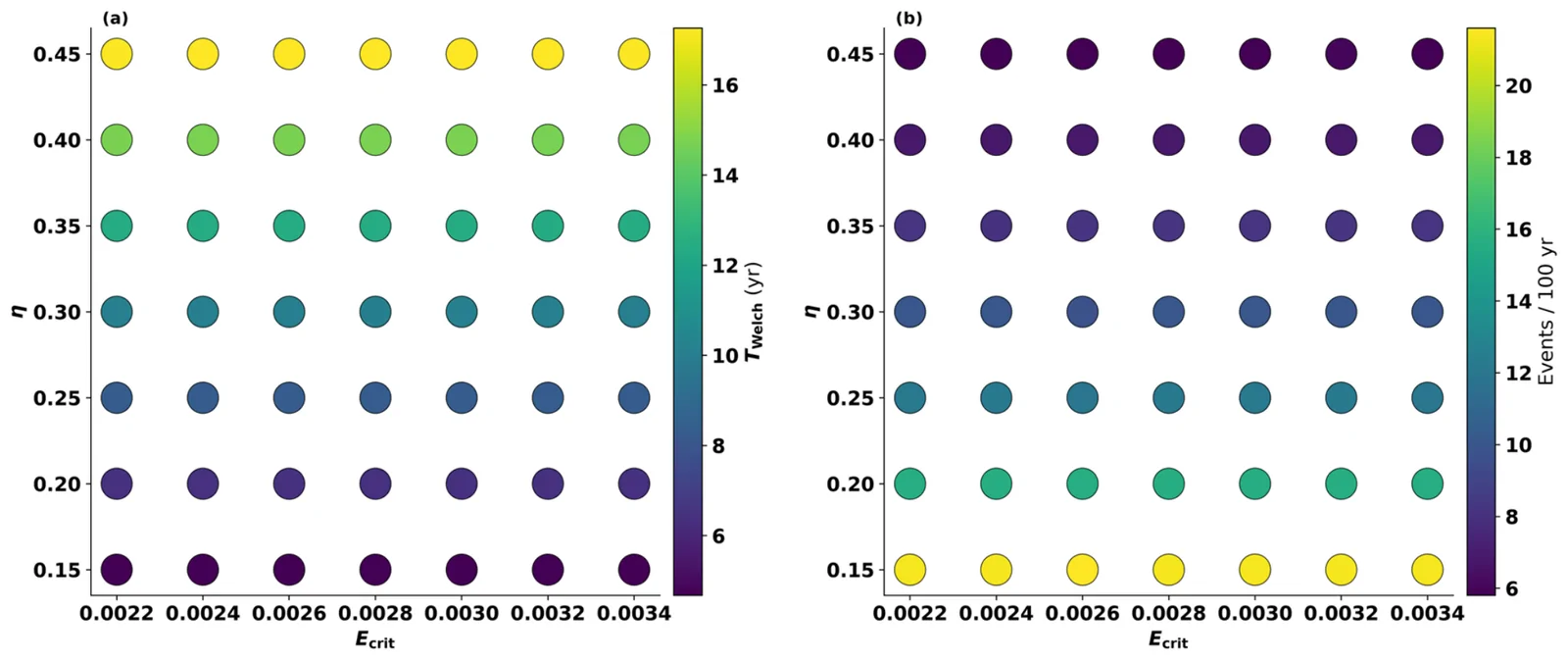
Robustness of the hybrid regime in the $E_{\mathrm{crit}}$ – η parameter plane. (**a**) Dominant collective Welch period and (**b**) release rate as functions of the critical magnetic energy $E_{\mathrm{crit}}$ and release depth η. Both quantities depend primarily on η, while showing comparatively weak sensitivity to $E_{\mathrm{crit}}$ over the tested range.

**Figure A9:**
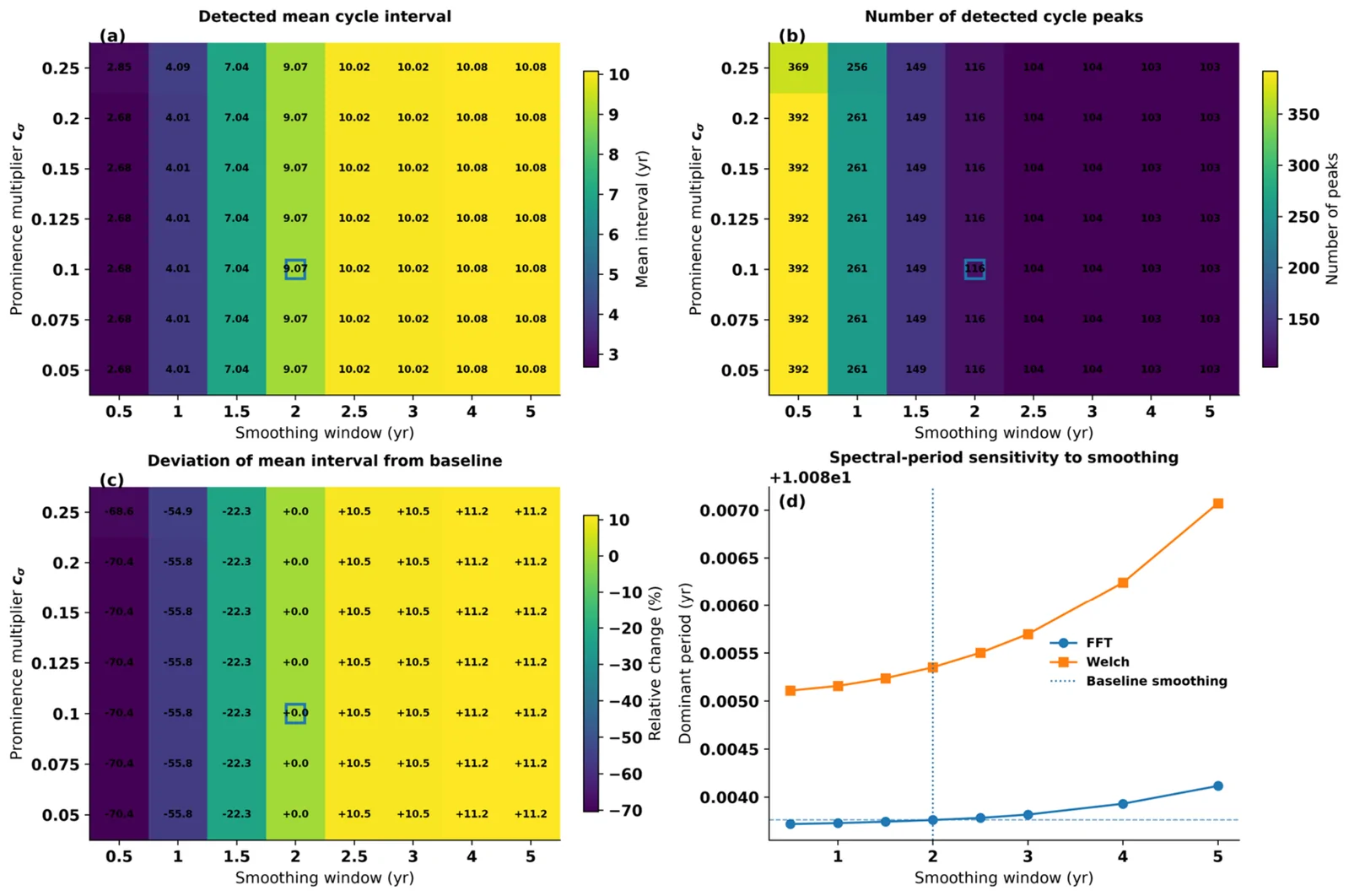
Post-processing sensitivity of the inferred collective cycle. Dependence of (**a**) the mean detected cycle interval, (**b**) the number of detected cycle peaks, and (**c**) the relative deviation of the mean interval from the baseline on smoothing window and prominence multiplier. (**d**) Corresponding FFT and Welch dominant periods.

**Figure A10:**
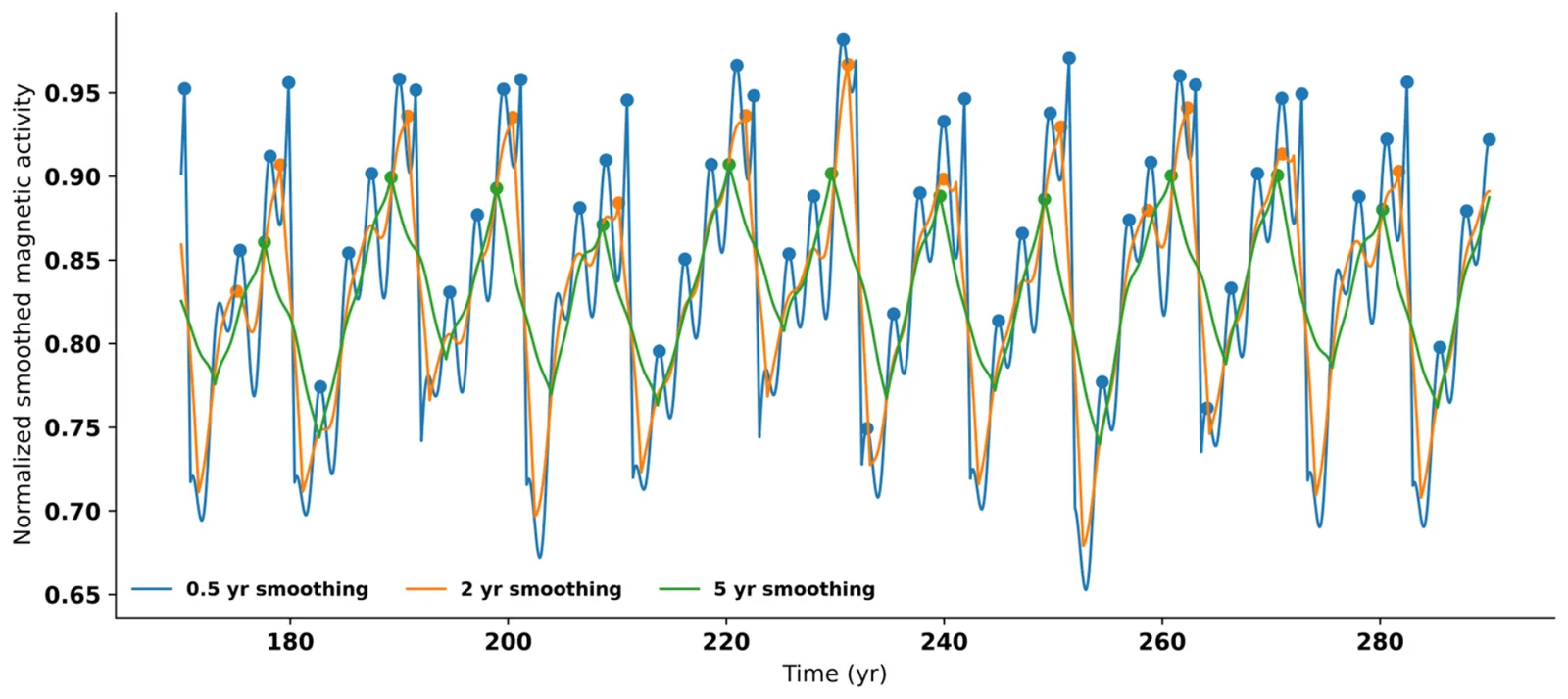
Representative cycle identification under different smoothing windows. Magnetic-activity trajectories processed using smoothing windows of 0.5, 2, and 5 yr.

**Figure A11:**
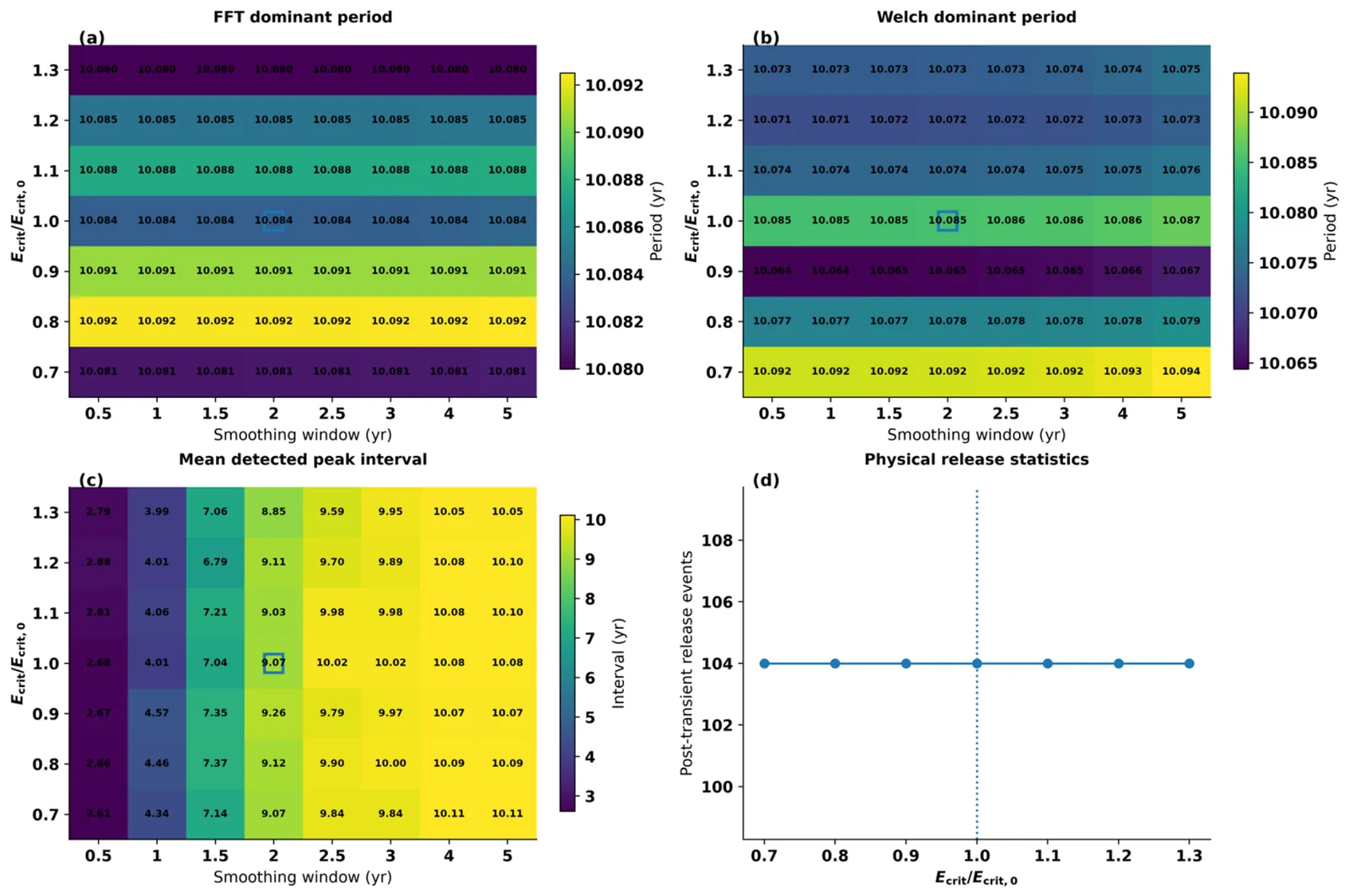
Robustness to the physical release threshold and smoothing. Dominant (**a**) FFT and (**b**) Welch periods as functions of normalized threshold $E_{\mathrm{crit}}/E_{\mathrm{crit},0}$ and smoothing-window length. (**c**) Mean interval between detected local activity peaks. (**d**) Number of post-transient physical hybrid release events as a function of $E_{\mathrm{crit}}/E_{\mathrm{crit},0}$.

## Data Availability

Data is contained within the article.

## Appendix A. Statistical Properties of Energy-Release Events

The hybrid formulation defines magnetic-energy release events directly through upward crossings of the critical-energy surface,

(A1) $$E_{3}(t_{k}^{-})=E_{\mathrm{crit}},\;\;{\dot{E}}_{3}(t_{k}^{-})>0,$$

where $t_{k}$ denotes the time of the k-th release event. This event-based definition makes it possible to characterize the temporal statistics of the release process without introducing an additional peak-detection threshold. For the baseline simulation, 116 release events were detected over the full 1200 yr integration, of which 104 occurred after removal of the initial 150 yr transient. Only the post-transient events were used for the statistical analysis presented below.

### Appendix A.1. Waiting-Time Statistics

The recurrence interval between consecutive release events was defined as

(A2) $$\Delta t_{k}=t_{k}-t_{k-1}.$$

The resulting waiting-time sequence exhibits fluctuations around a well-defined decadal characteristic timescale (Figure A1a). The mean waiting time is approximately $\langle \Delta t\rangle ≃10.08\mathrm{yr}$, while the median is approximately median(Δt)≃9.64yr.

The standard deviation is approximately 0.93 yr, giving a coefficient of variation

(A3) $$\mathrm{CV}=\frac{\sigma_{\Delta t}}{\langle \Delta t\rangle }≃0.093.$$

Thus, the release sequence exhibits finite event-to-event variability, but its dispersion is small compared with the characteristic recurrence interval.

The uncertainty of the mean waiting time was estimated using bootstrap resampling. The resulting 95% confidence interval is approximately $9.89\mathrm{yr}≲\langle \Delta t\rangle ≲10.27\mathrm{yr}$.

The waiting-time distribution exhibits two visually distinguishable concentrations rather than a simple symmetric unimodal shape (Figure A1b). Accordingly, the mean waiting time should not be regarded as a complete description of the recurrence statistics. For the post-transient events, the mean waiting time is approximately 10.08 yr, while the median is 9.64 yr and the standard deviation is 0.93 yr. The mean is retained as a convenient measure for comparison with the approximately decadal spectral timescale, whereas the median and dispersion provide complementary information on the event-recurrence structure. The apparent two-group structure is not interpreted here as statistically established bimodality, since no dedicated multimodality test was applied.

For the baseline hybrid regime, the mean waiting time is close to the independently estimated dominant spectral period, $T_{\mathrm{Welch}}≃10.09\mathrm{yr}$, yielding $T_{\mathrm{Welch}}\approx \langle \Delta t_{\mathrm{release}}\rangle$.

This correspondence supports the association of the dominant spectral timescale with the characteristic magnetic-energy recharge interval. As demonstrated by the event-skipping regime in the main text, however, this relation is characteristic of the regular baseline regime and is not imposed as a general constraint of the hybrid model.

### Appendix A.2. Tachocline-Phase Dependence

To determine whether threshold-triggered release events occur independently of the tachocline dynamics, the phase of the $x_{2}$ mode was evaluated at each post-transient release time. The resulting circular distribution is shown in Figure A1c.

The phases are not uniformly distributed over the oscillation cycle. The circular mean is approximately ${\bar{\phi }}_{2}≃4.35\mathrm{rad}≃249{}^{\circ},$ and the mean resultant length is R≃0.457.

The nonuniformity was tested using the Rayleigh statistic, yielding $p≃3.79\times 10^{-10}$.

The uniform-phase hypothesis is therefore strongly rejected, demonstrating that magnetic-energy release occurs preferentially within a particular phase sector of the tachocline mode.

The meant resultant length remains substantially below unity, however, indicating considerable dispersion of the event phases around the preferred direction. The result therefore does not imply strict phase locking between the tachocline oscillation and magnetic-energy release. Rather, the tachocline phase modulates the conditions under which the magnetic subsystem reaches the critical-energy surface.

This interpretation is consistent with the parameter analysis in the main text: the tachocline period does not generally prescribe the collective timescale, but tachocline dynamics can influence the timing and organization of threshold crossings. The phase statistics therefore support a picture in which the tachocline acts as a dynamical modulator of the accumulation–release process rather than as a rigid clock for individual release events.

## Appendix B. Additional Parameter Robustness Tests

To complement the intrinsic-period sensitivity analysis presented in Section 3.5 and Section 3.6, additional parameter tests were performed to determine whether the persistent decadal regime depends critically on the particular baseline parameter set. Two complementary analyses were considered. First, the critical magnetic-energy threshold $E_{\mathrm{crit}}$ and release depth η were varied jointly. Second, the coupling coefficients $k_{12}$ and $k_{23}$, magnetic self-excitation parameter μ, and magnetic Duffing coefficient $\alpha_{3}$ were varied independently around their baseline values.

### Appendix B.1. Robustness in the $E_{\mathrm{crit}}$ − η Parameter Plane

The hybrid release mechanism contains two parameters with distinct physical roles. The threshold $E_{\mathrm{crit}}$ specifies the magnetic-energy level at which a release event is triggered, whereas η determines the fractional reduction of magnetic energy during the event,

(A4) $$E_{3}^{+}=(1-\eta )E_{3}^{-}$$

Since release occurs at $E_{3}^{-}=E_{\mathrm{crit}}$, the post-release energy deficit is

(A5) $$\Delta E_{\mathrm{recharge}}=E_{\mathrm{crit}}-E_{3}^{+}=\eta E_{\mathrm{crit}}.$$

The joint parameter scan shows that the dominant Welch period is organized primarily along the η direction (Figure A2a). Increasing η systematically shifts the system toward longer characteristic periods, whereas variation in $E_{\mathrm{crit}}$ over the investigated range produces substantially smaller changes at fixed η. At the baseline value η = 0.30, for example, varying $E_{\mathrm{crit}}$ from approximately 0.0022 to 0.0034 changes the dominant period only from approximately 10.07 to 10.10 yr.

The release-rate map exhibits the complementary behavior (Figure A2b). Larger values of η generally produce lower release rates because the magnetic subsystem is transferred farther below the critical-energy surface after each event and consequently requires a longer recovery interval.

Importantly, the decadal regime is not restricted to a single finely tuned combination of $E_{\mathrm{crit}}$ and η. A finite region of the parameter plane produces characteristic periods in the solar-like decadal range. In the investigated domain, this region is approximately centered around 0.27≲η≲0.34, with comparatively weak sensitivity to the precise value of $E_{\mathrm{crit}}$.

These results indicate that $E_{\mathrm{crit}}$ and η play dynamically different roles. The former defines the triggering level, whereas the latter strongly affects the recovery distance following an event and therefore provides an important control of the recurrence timescale.

### Appendix B.2. Robustness to Coupling and Magnetic-Mode Parameters

A second sensitivity analysis was performed for four parameters governing the continuous dynamics: the coupling coefficients $k_{12}$ and $k_{23}$, the magnetic self-excitation coefficient μ, and the magnetic Duffing coefficient $\alpha_{3}.$ Each parameter was varied independently from 0.7 to 1.3 times its baseline value while the remaining parameters were held fixed.

The dominant period is only weakly affected by variations in the two linear coupling coefficients over the investigated range (Figure A3). Variation in $k_{12}$ produces Welch periods of approximately 10.08–10.19 yr, while variation in $k_{23}$ gives a similarly narrow interval, 10.08–10.19 yr.

Thus, ±30% changes in either coupling coefficient do not destroy the decadal regime and produce only modest shifts in its characteristic timescale.

The magnetic Duffing coefficient $\alpha_{3}$ has an even weaker effect. Across the investigated ±30% interval, the dominant period remains approximately $T_{\mathrm{Welch}}≃10.10\mathrm{yr}$, with a total variation of less than 0.01 yr. Within this parameter range, the nonlinear magnetic stiffness therefore affects the characteristic timescale only weakly.

A qualitatively different behavior is obtained for the magnetic self-excitation parameter μ. Increasing μ systematically decreases both the dominant spectral period and the mean waiting time between release events. Representative results are in Table A1.

**Table A1:** The mean waiting time and the dominant spectral period between release events.

$\mu /\mu_{0}$	$T_{Welch}$
0.7	14.69
0.8	12.83
0.9	11.36
1.0	10.10
1.1	9.29
1.2	8.54
1.3	7.88

where $\mu_{0}$ denotes the baseline value. The mean release interval follows essentially the same trend.

This strong sensitivity has a natural interpretation within the accumulation–release picture. The parameter η determines the energy deficit generated by a release,

(A6) $$\Delta E_{\mathrm{recharge}}=\eta E_{\mathrm{crit}}$$

whereas μ controls the self-excited dynamics of the magnetic subsystem and therefore strongly influences the rate at which this deficit is recovered. Consequently, the characteristic recurrence timescale may be represented schematically as

(A7) $$T_{\mathrm{collective}}\sim T_{\mathrm{recharge}}=F\left(\eta ,\mu ;T_{1},T_{2},T_{3},k_{12},k_{23},E_{\mathrm{crit}},\alpha_{i},\beta ,\ldots \right).$$

This expression is not intended as an analytical scaling law, but rather summarizes the hierarchy of sensitivities observed numerically.

The combined robustness tests therefore reveal an important distinction between structural parameters and timescale-controlling parameters. Over the investigated ranges, moderate variations in $k_{12}$, $k_{23}$, and $\alpha_{3}$ leave the decadal regime largely unchanged, whereas μ and η strongly modify its characteristic timescale. The decadal behavior is therefore robust to several details of the coupling structure but remains physically controlled by the accumulation and recovery properties of the magnetic subsystem.

## Appendix C. Energetic Diagnostics and Numerical Validation

The energetic and numerical consistency of the hybrid formulation was examined independently of its spectral and statistical properties. Three complementary tests were performed: analysis of the continuous-flow magnetic-energy exchange, verification of the discrete reset operator, and conservation of the total energy in the conservative limit. These tests distinguish the continuous dynamics from the instantaneous energy change associated with threshold-triggered release events.

### Appendix C.1. Continuous-Flow Structural Energy-Exchange Diagnostic

The magnetic subsystem energy is defined as

(A8) $$E_{3}=\frac{1}{2}v_{3}^{2}+\frac{1}{2}\omega_{3}^{2}x_{3}^{2}+\frac{1}{4}\alpha_{3}x_{3}^{4}.$$

Along continuous segments of the hybrid trajectory, its rate of change is

(A9) $${\left(\frac{dE_{3}}{dt}\right)}_{\mathrm{flow}}=v_{3}{\dot{v}}_{3}+\left(\omega_{3}^{2}x_{3}+\alpha_{3}x_{3}^{3}\right){\dot{x}}_{3}.$$

The corresponding structural energy-exchange diagnostic was evaluated as

(A10) $$I_{\mathrm{str}}^{\mathrm{flow}}=\frac{1}{E_{3}}{\left(\frac{dE_{3}}{dt}\right)}_{\mathrm{flow}}.$$

This quantity characterizes the relative instantaneous rate of magnetic-energy change generated by the continuous dynamics. Positive values, $I_{\mathrm{str}}^{\mathrm{flow}}>0$, correspond to intervals of net magnetic-energy growth, whereas negative values indicate net energy loss.

The baseline hybrid trajectory exhibits repeated accumulation of $E_{3}$ toward the critical level $E_{\mathrm{crit}}$, followed by an instantaneous reduction at each release event (Figure A4a). The corresponding $I_{\mathrm{str}}^{\mathrm{flow}}$ displays alternating positive and negative intervals associated with continuous energy redistribution among the coupled modes (Figure A4b).

An important distinction is required at the reset times. Because the hybrid release is represented by a discontinuous state transformation, the discrete jump should not be included in a conventional time derivative. Accordingly, $I_{\mathrm{str}}^{\mathrm{flow}}$ is evaluated only between release events. The energy removed during a reset is treated separately through the discrete energy jump considered in Appendix C.2.

This separation prevents the instantaneous reset from producing artificial derivative spikes and provides a consistent distinction between continuous structural energy exchange and discrete threshold-triggered energy release.

### Appendix C.2. Numerical Accuracy and Energetics of the Hybrid Reset

At each upward crossing of the critical-energy surface, $E_{3}(t_{k}^{-})=E_{\mathrm{crit}}$, the magnetic state is transformed according to

(A11) $$x_{3}^{+}=qx_{3}^{-},\;\;v_{3}^{+}=qv_{3}^{-}$$

where the scaling factor q∈(0,1) is determined numerically by Equation (A4).

The reset accuracy was evaluated for all 116 release events detected during the baseline 1200 yr simulation. The relative reset error was defined as

(A12) $$\epsilon_{k}=\frac{\left|E_{3,k}^{+}-(1-\eta )E_{3,k}^{-}\right|}{(1-\eta )E_{3,k}^{-}}.$$

The reset condition is reproduced to near machine precision (Figure A5a). The maximum relative error is $\max(\epsilon_{k})=4.13\times 10^{-16}$, while the median value is $\mathrm{median}(\epsilon_{k})=2.07\times 10^{-16}$.

These values demonstrate that the numerical root-finding procedure used to determine q introduces no dynamically significant error.

For the baseline parameters $E_{\mathrm{crit}}$ = 0.003, η = 0.30, the magnetic energy immediately after release is $E_{3}^{+}$ = 0.0021, and the intrinsic magnetic-energy reduction is therefore $\Delta E_{3}=E_{3}^{-}-E_{3}^{+}=9.0\times 10^{-4}$.

Consequently, $\Delta E_{3}$ remains essentially constant from event to event because both $E_{\mathrm{crit}}$ and η are fixed.

The change in the total system energy is not required to equal $\Delta E_{3}$. The total energy contains interaction terms,

(A13) $$E_{\mathrm{tot}}=E_{1}+E_{2}+E_{3}+V_{12}+V_{23}+V_{\beta }$$

with

(A14)
$$V_{12}=\frac{k_{12}}{2}{\left(x_{2}-x_{1}\right)}^{2},\;\;V_{23}=\frac{k_{23}}{2}{\left(x_{3}-x_{2}\right)}^{2},\;\;V_{\beta }=-\beta x_{2}^{2}x_{3}.$$

Because the reset changes $x_{3}$, it also changes these interaction energies. The total energy removed during the k-th event is therefore

(A15) $$\Delta E_{\mathrm{tot},k}=E_{\mathrm{tot}}(t_{k}^{-})-E_{\mathrm{tot}}(t_{k}^{+}),$$

and exhibits small event-to-event variations despite the fixed value of $\Delta E_{3}$ (Figure A5b).

The distinction between $\Delta E_{3}$ and $\Delta E_{\mathrm{tot}}$ is therefore a direct consequence of applying the reset to a subsystem that remains coupled to the rest of the system, rather than a numerical inconsistency.

### Appendix C.3. Energy Conservation in the Conservative Limit

A separate conservative-limit experiment was performed to verify the implementation of the reciprocal coupling terms. Dissipation, magnetic self-excitation, and threshold-triggered release were removed, leaving only the conservative modal dynamics and interaction potentials.

The corresponding total energy is Equation (A13). The nonlinear interaction potential generates the reciprocal forces

(A16) $$\begin{array}{l} F_{2,\beta }=-\frac{\partial V_{\beta }}{\partial x_{2}}=2\beta x_{2}x_{3},\\ F_{3,\beta }=-\frac{\partial V_{\beta }}{\partial x_{3}}=\beta x_{2}^{2}. \end{array}$$

This reciprocal structure is necessary for the nonlinear interaction to remain conservative.

Numerical conservation was quantified using the relative energy drift

(A17) $$\delta E_{\mathrm{rel}}(t)=\frac{E_{\mathrm{tot}}(t)-E_{\mathrm{tot}}(0)}{E_{\mathrm{tot}}(0)}.$$

Over the validation trajectory, the maximum absolute relative drift was $\max_{t}\left|\delta E_{\mathrm{rel}}(t)\right|=5.67\times 10^{-15}$.

The total energy therefore remains constant to near machine precision, with no systematic secular drift (Figure A6). This result provides an independent numerical verification that the reciprocal linear and nonlinear coupling terms and the associated interaction potentials have been implemented consistently.

The conservative-limit test is particularly important because the full hybrid model is intentionally nonconservative: continuous dissipation and self-excitation modify the subsystem energies, while discrete release events remove energy through the reset operator. Energy conservation is therefore not expected for the complete model. Instead, the conservative-limit experiment isolates the underlying interaction structure and verifies that energy nonconservation in the full simulation originates from explicitly prescribed nonconservative mechanisms rather than from an inconsistency in the coupling formulation.

## Appendix D. Event-Based Dynamical Diagnostics

Because the proposed model combines continuous nonlinear evolution with discrete threshold-triggered resets, conventional diagnostics developed exclusively for smooth dynamical systems require additional care. In particular, the release events introduce discontinuities in the trajectory and naturally define an event-based representation of the dynamics. We therefore complement the continuous-time analysis with diagnostics constructed from the system state immediately before each magnetic-energy release and from the recurrence intervals between successive events.

### Appendix D.1. Release-Event Phase-Space Section

Each magnetic-energy release occurs when the trajectory reaches the threshold surface Equation (A1). The sequence of pre-release states therefore provides a natural event-defined section of the hybrid dynamics. To examine the state of the intermediate subsystem at these crossings, the tachocline variables were sampled immediately before each reset,

(A18) $$z_{k}=\left(x_{2}(t_{k}^{-}),v_{2}(t_{k}^{-})\right)$$

The resulting points in the ($x_{2}$, $v_{2}$) plane form a structured distribution rather than filling the accessible phase-space region uniformly (Figure A7a). Thus, although all events satisfy the same magnetic-energy threshold condition, they occur for different instantaneous states of the tachocline subsystem.

This result is consistent with the phase statistics presented in Appendix A. Release events exhibit a statistically significant preference for a particular tachocline phase sector, but they are not restricted to a single phase. The event section provides a complementary phase-space representation of this behavior: threshold crossing is dynamically organized by the coupled system, while retaining finite variability between successive events.

The event-defined section should not be interpreted as a conventional Poincaré section of a purely smooth autonomous flow. Each threshold crossing is followed by the reset transformation

(A19) $$y_{k}^{+}=R(y_{k}^{-}),$$

after which the continuous evolution resumes. The complete event-to-event dynamics therefore consists of both continuous propagation and the discrete reset map.

Accordingly, the structure observed in Figure A7a is used here as a diagnostic of the organization of release states rather than as evidence for a specific attractor topology.

### Appendix D.2. Waiting-Time Recurrence Map

The temporal organization of successive release events was further examined using the recurrence map $\Delta t_{k}\to \Delta t_{k+1},$ where $\Delta t_{k}=t_{k}-t_{k-1}$.

If the release process were exactly periodic, all points would collapse onto a single location,

(A20) $$\Delta t_{k}=\Delta t_{k+1}=T_{\mathrm{release}}.$$

Instead, the recurrence map exhibits a finite distribution around the characteristic decadal recurrence scale (Figure A7b). This is consistent with the nonzero waiting-time dispersion reported in Appendix A, 〈Δt〉≃10.08yr, CV≃0.093.

The finite spread confirms that successive threshold crossings are recurrent but not identically periodic. At the same time, the points remain confined to a relatively restricted region of the recurrence plane, consistent with the persistent long-term accumulation–release regime identified from the spectral analysis.

The recurrence map therefore provides an event-based complement to the waiting-time distribution. Whereas the latter describes the marginal statistics of $\Delta t_{k}$, the recurrence representation retains information about the ordering of consecutive intervals and can reveal event-to-event organization that is not apparent from the distribution alone.

### Appendix D.3. Local Cycle-to-Cycle and Release-Interval Variability

The similarity between the dominant spectral period and the mean release interval in the baseline regime raises the question of whether individual activity cycles are directly synchronized with individual release intervals. To test this possibility, local activity-cycle durations were compared with neighboring intervals between successive magnetic-energy release events (Figure A8).

No statistically significant local association was detected. The Pearson correlation coefficient was r = −0.106, p = 0.288, while the Spearman rank correlation coefficient was ρ = 0.074, p = 0.456.

Both tests therefore indicate that fluctuations in individual cycle durations do not systematically correspond to fluctuations in neighboring release intervals.

This result is important for interpreting the approximate global relation

(A21) $$T_{\mathrm{spectral}}\approx \langle \Delta t_{\mathrm{release}}\rangle .$$

The agreement between these quantities characterizes the long-term statistical organization of the regular hybrid regime; it does not imply a one-to-one correspondence between individual oscillatory cycles and individual threshold crossings.

The two quantities represent different dynamical observables. A local activity-cycle duration is determined from the continuous oscillatory evolution of the magnetic subsystem, whereas a release interval is determined by successive intersections of the full coupled trajectory with the threshold surface $E_{3}=E_{\mathrm{crit}}$.

Consequently, the timing of a release depends not only on the instantaneous magnetic oscillation but also on the coupled state of the other modes and on the accumulated magnetic energy.

This distinction becomes particularly clear in the event-skipping regime described in Section 3.6. There, an oscillatory cycle may occur without reaching $E_{\mathrm{crit}}$, allowing more than one oscillatory excursion between successive release events. The spectral period and release interval can consequently separate substantially.

The absence of significant local correlation therefore does not contradict their similar characteristic values in the baseline regime. Instead, it shows that the decadal spectral and recurrence timescales are statistically related manifestations of the same coupled hybrid dynamics rather than strictly synchronized event-by-event quantities.

## Appendix E. Post-Processing Sensitivity of Cycle Identification

### Appendix E.1. Sensitivity to Smoothing and Peak Detection

To determine whether the inferred collective timescale depends on the post-processing procedure, the validated baseline trajectory was analyzed repeatedly while keeping the physical model unchanged. No dynamical parameter, release threshold $E_{\mathrm{crit}}$, or reset parameter was modified. Only the smoothing window and the prominence threshold used for local peak detection were varied. The tested smoothing windows were in the range 0.5≤w≤5 yr, while the prominence multiplier was in the range $0.05\leq c_{\sigma }\leq 0.25,$ resulting in 56 post-processing configurations. The baseline settings were w = 2 yr and $c_{\sigma }=0.10$, with a prominence floor of 0.02 and a minimum peak distance of 1 yr.

Under the baseline settings, 116 local cycle peaks were detected, giving a mean peak-to-peak interval of $\langle T_{\mathrm{peak}}\rangle =9.067$ yr, whereas the independently determined spectral periods were $T_{\mathrm{FFT}}$ = 10.084 yr, $T_{\mathrm{Welch}}$ = 10.085 yr.

The sensitivity map in Figure A9a–c shows that the identification of individual local maxima depends strongly on the adopted smoothing scale. Across the complete grid, the number of detected peaks varied from 103 to 392, while the mean peak-to-peak interval changed by as much as 70.4% relative to the baseline value.

In contrast, the dominant spectral timescale was essentially unaffected by the post-processing parameters. As shown in Figure A9d, the maximum relative changes in the FFT and Welch estimates were only 0.004% and 0.017%, respectively. Thus, the approximately decadal spectral component is not generated by the chosen smoothing or prominence threshold.

The origin of the peak-count sensitivity is illustrated directly in Figure A10.

At short smoothing windows, secondary local maxima remain visible and are counted as separate peaks, whereas stronger smoothing suppresses these short-timescale structures and retains only the broader modulation. The detected local cycle sequence is therefore post-processing-dependent even though the long-term spectral organization remains practically unchanged.

These results motivate a strict distinction between the dominant collective spectral timescale and intervals obtained from local peak detection. In the present study, $T_{\mathrm{collective}}\equiv T_{\mathrm{Welch}}$ is used as the primary estimate of the long-term collective timescale, whereas peak-to-peak intervals are retained only as complementary measures of local cycle variability.

### Appendix E.2. Combined Sensitivity to the Physical Threshold and Smoothing

A second robustness experiment was performed to separate the effects of a physical hybrid-model parameter from those introduced by post-processing. The magnetic-energy threshold $E_{\mathrm{crit}}$ was varied over $0.7\leq \frac{E_{\mathrm{crit}}}{E_{\mathrm{crit},0}}\leq 1.3,$ where $E_{\mathrm{crit},0}=0.003,$ while the smoothing window was varied independently between 0.5 and 5 yr. Unlike the smoothing operation, $E_{\mathrm{crit}}$ directly modifies the hybrid dynamics; therefore, the trajectory was re-integrated independently for each threshold value before smoothing was applied.

The resulting spectral periods are shown in Figure A11a,b. Across the complete $E_{\mathrm{crit}}$-smoothing grid, the FFT period remained within $10.080\leq T_{\mathrm{FFT}}\leq 10.093$ yr, and the Welch estimate remained within $10.064\leq T_{\mathrm{Welch}}\leq 10.094$ yr.

Hence, even simultaneous variation of the physical threshold and the post-processing scale produces only a very small change in the inferred decadal spectral timescale.

The behavior of the peak-based interval is substantially different. As shown in Figure A11c, the mean detected peak interval ranges from approximately 2.61 yr to 10.11 yr over the full grid. The lowest values occur for short smoothing windows, where secondary maxima are retained. When the smoothing window is restricted to w≥2 yr, the range narrows considerably to approximately 8.85–10.11 yr.

This confirms that most of the variability in the peak-based interval is associated with local peak identification rather than with changes in the long-term collective spectral scale.

A particularly important result is shown in Figure A11d. The number of post-transient physical release events remains equal to $N_{\mathrm{release}}=104$ for all tested values of $E_{\mathrm{crit}}/E_{\mathrm{crit},0}$. Thus, within the investigated threshold neighborhood, variation in $E_{\mathrm{crit}}$ modifies neither the number of post-transient hybrid releases nor the overall decadal organization of the trajectory.

The combined test therefore separates three distinct quantities: the dominant spectral period, the physical hybrid release sequence, and the local peak sequence extracted from a smoothed activity observable. The first two are determined by the model dynamics, whereas the third is more sensitive to post-processing choices. Consequently, local maxima should not be used as the primary definition of the collective cycle.

## Appendix F. Extended Validation of the Hybrid Lyapunov Analysis

### Appendix F.1. Finite-Difference Validation of the Reset Jacobian

The hybrid Lyapunov analysis requires the differential of the nonlinear reset map entering the saltation matrix. Because the reset factor q is determined implicitly from the prescribed post-release magnetic-energy condition, the reset Jacobian contains the state dependence of q. To verify the analytical implementation independently, the complete reset Jacobian DR was compared with a central finite-difference approximation evaluated directly from the nonlinear reset map.

For a pre-event magnetic state

(A22) $$z={\left(x_{3},v_{3}\right)}^{T},$$

the reset is defined as

(A23) $$R_{z}(z)=q(z)z,$$

where q(z) satisfies

(A24) $$E_{3}(qz)=(1-\eta )E_{3}(z).$$

The analytical magnetic block of the reset Jacobian is

(A25) $$DR_{z}=qI+z{\left(\nabla q\right)}^{T}.$$

An independent central finite-difference approximation was calculated as

(A26) $${\left(DR_{\mathrm{FD}}\right)}_{ij}=\frac{R_{i}(y+\delta_{j}e_{j})-R_{i}(y-\delta_{j}e_{j})}{2\delta_{j}},$$

where the nonlinear reset factor was recomputed independently for each perturbed state. The comparison was performed at 20 actual upward threshold crossings of the baseline hybrid trajectory. The relative discrepancy was quantified using

(A27) $$\varepsilon_{DR}=\frac{\|DR-DR_{\mathrm{FD}}{\|}_{F}}{\|DR{\|}_{F}}.$$

The relative finite-difference step was varied over several orders of magnitude rather than selecting a single perturbation amplitude. As shown in Figure A12, the resulting errors exhibit the characteristic finite-difference convergence pattern: errors decrease as the perturbation step is reduced from large values, reach a minimum at intermediate step sizes, and increase again for the smallest steps because of floating-point cancellation and round-off effects.

Across the 20 tested threshold crossings, the median best relative Frobenius error was $6.73\times 10^{-13}$, and the maximum best relative error was $1.20\times 10^{-12}$.

In addition, the maximum relative error in satisfying the prescribed post-reset magnetic-energy condition was $4.13\times 10^{-16}$.

These results provide an independent numerical verification of the analytical state-dependent reset Jacobian used in the saltation matrix and confirm that the derivatives of the implicit reset factor q are correctly incorporated in the hybrid variational calculation.

### Appendix F.2. Extended-Horizon Validation of Positive Finite-Time Lyapunov Estimates

The baseline convergence analysis demonstrated that the sign of a small finite-time largest Lyapunov exponent can depend on the accumulation horizon. Therefore, positive values obtained in the 1000 yr parameter sweep (Figure 8) were subjected to an additional extended-horizon test rather than being interpreted directly as evidence of asymptotic chaos.

All 13 one-factor parameter configurations for which $\lambda_{\max}(1000)>0$ were selected and recomputed using a 1500 yr post-transient accumulation horizon while retaining the same hybrid variational formulation, state-dependent reset Jacobian, saltation correction, numerical integration settings, and QR-renormalization procedure.

The comparison is shown in Figure A13. Of the 13 configurations selected from the 1000 yr sweep, 10 retained positive finite-time largest Lyapunov estimates at 1500 yr, whereas three changed sign. Persistent positive estimates were obtained for four tested variations of $k_{12}$, two variations of $k_{23}$, two values of the release depth η, one value of μ, and one value of $E_{\mathrm{crit}}$.

The behavior is not uniform across the selected cases. For example, for $k_{23}$ = 0.112, the estimate remained positive and changed only moderately, $\lambda_{\max}:1.9925\times 10^{-3}\to 2.0484\times 10^{-3}\;{\mathrm{yr}}^{-1}$, when the accumulation horizon was increased from 1000 to 1500 yr. Similarly, for $E_{\mathrm{crit}}$ = 0.0039, the estimate remained positive, $1.0968\times 10^{-3}\to 9.6397\times 10^{-4}\;{\mathrm{yr}}^{-1}$.

In contrast, some smaller positive 1000 yr estimates did not persist at the longer horizon and became negative at 1500 yr.

No near-grazing threshold crossings were detected in the extended calculations. The observed sign changes therefore cannot be attributed to numerical singularities associated with grazing of the event surface.

These results reinforce the need to distinguish finite-time exponential sensitivity from an asymptotic dynamical classification. The baseline regime does not retain a positive largest Lyapunov exponent as the accumulation horizon is increased, whereas several neighboring parameter configurations preserve positive finite-time estimates up to 1500 yr. We therefore interpret these configurations as exhibiting persistent positive finite-time exponential sensitivity, without identifying them categorically as asymptotically chaotic regimes. A definitive asymptotic classification would require substantially longer integrations and a dedicated hybrid bifurcation analysis.
